# Elucidation of the Bovine Intramammary Bacteriome and Resistome from healthy cows of Swiss dairy farms in the Canton Tessin

**DOI:** 10.3389/fmicb.2023.1183018

**Published:** 2023-07-31

**Authors:** Alicia Romanò, Ivana Ivanovic, Tina Segessemann, Laura Vazquez Rojo, Jérôme Widmer, Lotti Egger, Matthias Dreier, Lorenzo Sesso, Michael Vaccani, Martin Schuler, Daniel Frei, Juerg Frey, Christian H. Ahrens, Adrian Steiner, Hans Ulrich Graber

**Affiliations:** ^1^Food Microbial Systems, Group Microbiological Safety of Foods of Animal Origin, Agroscope, Bern, Switzerland; ^2^Graduate School of Cellular and Biomedical Sciences, University of Bern, Bern, Switzerland; ^3^SIB, Swiss Institute of Bioinformatics, Zürich, Switzerland; ^4^Method Development and Analytics, Group Molecular Ecology, Agroscope, Zürich, Switzerland; ^5^Method Development and Analytics, Group Biochemistry of Milk, Agroscope, Bern, Switzerland; ^6^Food Microbial Systems, Group Cultures, Biodiversity, and Terroir, Agroscope, Bern, Switzerland; ^7^Clinic of Ruminants, Vetsuisse Faculty, University of Bern, Bern, Switzerland; ^8^Method Development and Analytics, Group Molecular Diagnostics, Genomics, and Bioinformatics, Agroscope, Wädenswil, Switzerland

**Keywords:** One Health, mastitis, intramammary bacteria healthy cows, antimicrobial resistance genes, whole genome sequencing, type strains, plasmids, antibiotics

## Abstract

Healthy, untreated cows of nine dairy herds from the Swiss Canton Tessin were analyzed three times within one year to identify the most abundant species of the intramammary bacteriome. Aseptically collected milk samples were cultured and bacteria identified using MALDI-TOF. Of 256 cows analyzed, 96% were bacteriologically positive and 80% of the 1,024 quarters were positive for at least one bacterial species. 84.5% of the quarters were healthy with somatic cell counts (SCC) < 200,000 cells/mL, whereas 15.5% of the quarters showed a subclinical mastitis (SCC ≥ 200,000 cells/mL). We could assign 1,288 isolates to 104 different bacterial species including 23 predominant species. Non-*aureus* staphylococci and mammaliicocci (NASM) were most prevalent (14 different species; 73.5% quarters). *Staphylococcus xylosus* and *Mammaliicoccus sciuri* accounted for 74.7% of all NASM isolates. To describe the intramammary resistome, 350 isolates of the predominant species were selected and subjected to short-read whole genome sequencing (WGS) and phenotypic antibiotic resistance profiling. While complete genomes of eight type strains were available, the remaining 15 were *de novo* assembled with long reads as a resource for the community. The 23 complete genomes served for reference-based assembly of the Illumina WGS data. Both chromosomes and mobile genetic elements were examined for antibiotic resistance genes (ARGs) using in-house and online software tools. ARGs were then correlated with phenotypic antibiotic resistance data from minimum inhibitory concentration (MIC). Phenotypic and genomic antimicrobial resistance was isolate-specific. Resistance to clindamycin and oxacillin was most frequently observed (65 and 30%) in *Staphylococcus xylosus* but could not be linked to chromosomal or plasmid-borne ARGs. However, in several cases, the observed antimicrobial resistance could be explained by the presence of mobile genetic elements like *tetK* carried on small plasmids. This represents a possible mechanism of transfer between non-pathogenic bacteria and pathogens of the mammary gland within and between herds. The-to our knowledge-most extensive bacteriome reported and the first attempt to link it with the resistome promise to profoundly affect veterinary bacteriology in the future and are highly relevant in a One Health context, in particular for mastitis, the treatment of which still heavily relies on antibiotics.

## 1. Introduction

Bovine mastitis, also referred to as bovine intramammary infection (IMI) caused by pathogens, is the most important and costly disease of dairy cows worldwide (Seegers et al., 2003). Severe economic losses in dairy cattle herds are caused by four main factors: reduced milk yield, unsuitability of the milk for consumption, antibiotic treatment costs, and culling of animals in case of treatment failure (Ruegg, 2017). In the frame of the One Health concept, which advocates a general view of human, animal and environmental health, research aimed at describing the bacterial diversity of both commensals and potential pathogens in animal food production systems is highly relevant (Aslam et al., 2021). Such research is expected to considerably reduce the use of antibiotics and thereby the associated dangers of transfer of antibiotic resistance genes (ARGs) and spreading of multi-resistant strains (McEwen and Collignon, 2018). While IMI by pathogens is well understood, very little is known about the bacteria present in the mammary gland of healthy and untreated cows and their antimicrobial resistances (AMR) (= intramammary resistome, IR). A few studies of the milk microbiota, relying both on culture-dependent and culture-independent approaches have been carried out in recent years [reviewed in Quigley et al. (2013) and Oikonomou et al. (2020)]. They arrived at the preliminary conclusion that milk of healthy udders is not a sterile matrix, but instead, harbors a complex microbial community composed of different microorganisms (Addis et al., 2016).

In subclinical IMI, non-*aureus* staphylococci (NAS) and *Streptococcus uberis* are the most frequently isolated bacteria (De Visscher et al., 2015). Despite NAS being considered less pathogenic than *Staphylococcus aureus*, they can carry virulence factors, toxins and antibiotic resistance genes and are able to generate biofilms (Vanderhaeghen et al., 2014; Taponen et al., 2016). However, their potential pathogenicity needs to be further clarified and evaluated in more detail. Potentially, they could just represent commensal microorganisms of the normal flora in the mammary gland (De Buck et al., 2021). A recent Swiss bacteriome study by Sartori et al. (2018) reported that NAS were the main bacteria colonizing healthy cows with *Staphylococcus xylosus* and *Staphylococcus chromogenes* representing the most frequent isolates from the milk of the selected herds. However, the IR of healthy cows was not assessed in that study.

Only recently in 2020, five species belonging to the NAS were reclassified in the novel genus *Mammaliicoccus* due to an evolutionary study based on 16S sequencing conducted by Madhaiyan et al. (2020). Nowadays, the staphylococci and mammaliicocci are indicated with the acronym of non-*aureus* staphylococci and mammaliicocci (NASM) (Rosa et al., 2022).

In Switzerland, the application of antibiotics (AB) in agriculture has been decreasing over the last years (2010–2019), evidenced by a 52% reduction of the sales of ABs used in livestock animals since 2010 (Federal Office of Public Health and Federal Food Safety and Veterinary Office, 2020). In part, this reduction can be attributed to the fact that critical antibiotic classes for human medicine (fluoroquinolones, macrolides, and 3^rd^/4^th^ generation cephalosporins) were banned to be given for stocks due to the Ordinance on Veterinary Medicinal Product, in line with the aims of the One Health approach (Federal Office of Public Health and Federal Food Safety and Veterinary Office, 2018). However, in contrast to this overall reduction, the use of antimicrobials licensed for treatment of IMI was relatively stable during 2010–2019 (Switzerland in fact has the highest use of intramammary products in Europe) (European Medicines Agency, European Surveillance of Veterinary Antimicrobial Consumption, 2021). Overall, 70% of all antimicrobials administered concern antibiotics for the treatment of mastitis during lactation. The main antibiotic is penicillin followed by aminoglycosides and cephalosporins. This high rate of applications could represent an important reason for the development of resistances in pathogens inducing mastitis episodes and in bacteria colonizing the healthy mammary gland (Oliver and Murinda, 2012).

Unfortunately, at the European level only resistance data of NASM strains responsible for clinical mastitis are available which displayed a high resistance to penicillin G and oxacillin (29.1 and 43.9%, respectively) (El Garch et al., 2020). In contrast, no phenotypic resistance data are available regarding the intramammary bacteriome of healthy cows except for one Swiss study from Frey et al. (2013). In this study, NASM were isolated from control milk, i.e., milk from healthy cows previously positive to mastitis, subjected to treatment, with the characteristic to have somatic cell counts (SCC) <150,000 cells/mL. The results of this study showed a prevalence of phenotypic resistance between 17 to 40% to the antibiotics oxacillin, fusic acid, tiamulin, penicillin, tetracycline, and streptomycin. Increasing research on comparing the resistance profiles of isolates from different countries could provide relevant insights into treatment strategies of affected herds.

Advances in whole genome sequencing (WGS) and the availability of online tools supporting researchers in the identification of antimicrobial resistance genes (ARGs) are important pre-requisites for studying not only the abundance and dissemination of AMR (Hendriksen et al., 2019), but also the potential transfer of ARGs from species colonizing animals to species infecting humans (Wendlandt et al., 2015). The transfer of ARGs commonly involves mobile genetic elements (MGEs). The most prevalent ones are plasmids, i.e., extrachromosomal DNA molecules that encode genes that play a role, among others, in virulence, antibiotic resistance, tolerance to heavy metals, and metabolism of carbon sources (Malachowa and Deleo, 2010). The classification of plasmids based on the replicon protein (Rep) is an important approach that can be used to examine the distribution of such MGEs in the environment (Orlek et al., 2017).

In the present study, more than 1,200 bacterial isolates were identified allowing to describe 23 predominant species of the intramammary bacteriome of healthy cows. Furthermore, WGS and phenotypic profiling was carried out for 350 isolates from the 23 most abundant species to infer the resistome (IR) at the phenotypic and the gene level and attempt to link phenotype and genotype information. The results are discussed under the aspects of diagnostic and clinical importance as well as of the One Health approach.

## 2. Materials and methods

### 2.1. Study design and sample collection

Nine different herds were randomly selected in the Swiss Canton of Tessin. Quarter milk samples were collected aseptically 3 times during winter 2017–2018 (time point 0), late spring 2018 (time point 1, sampling was performed before the cows were sent for common pasturing on alps during the summer season), and winter 2018–2019 (time point 2) from at least 10 randomly selected lactating cows (unless stated otherwise; Table 1), following the guidelines of the National Mastitis Council [National Mastitis Council (NMC), 2016]. Prerequisites for inclusion in the study were that the cows (i) did not receive any antibiotic therapy within the previous five days, (ii) did not show any clinical signs of mastitis or teat injuries, (iii) appeared visually normal, and (iv) their milk was suitable for human consumption according to Swiss legislation (VHyMP 2020^1^). Data regarding age, lactation number, and stage of lactation of the cows were collected. Considering the stage of lactation, we referred to three different stages divided in early (14–100 days after calving), mid (100–200 days after calving), and late lactation (>200 days after calving). For lactation number, we divided the cows into three different groups: (i) 1st lactation (primiparous), (ii) 2nd and 3rd lactation, and (iii) >3 lactations. For herd 6, only samples from the 1st and 2nd sampling could be collected as the farm was given up later.

**Table 1:** Overview of the within-herd bacterial positivity for the three sampling time points.

Number of quarters positive per farm and sampling																											
	Farm 1			Farm 2			Farm 3			Farm 4			Farm 5			Farm 6		Farm 7			Farm 8			Farm 9			Total
Sampling	0	1	2	0	1	2	0	1	2	0	1	2	0	1	2	0	1	0	1	2	0	1	2	0	1	2	
Type of Bedding	Straw, sawdust			Straw, sawdust			Straw			Straw, ferus			Straw			Straw		Straw			Straw			Manure			
N. cow sampled	11	10	10	9	8	9	10	10	10	10	10	10	10	10	10	9	10	10	10	10	10	10	10	10	10	10	256
N. cow bacteriologically positive	11	10	10	9	8	8	10	10	10	10	10	7	10	10	9	9	10	10	10	10	10	10	9	10	10	10	250
N. quarter sampled	44	40	40	36	32	36	40	40	40	40	40	40	40	40	40	36	40	40	40	40	40	40	40	40	40	40	1,024
N. quarter bacteriologically positive	43	31	31	32	31	15	38	34	34	39	37	12	29	37	23	27	35	30	36	26	39	40	24	36	31	34	824
Quarters positive only with one bacterial species	18	24	18	4	22	6	15	17	18	26	15	10	19	14	15	17	15	20	18	17	20	15	18	21	19	19	440
Quarters positive with 2 bacterial species	24	5	10	23	8	7	17	11	12	11	13	1	9	17	6	8	17	10	15	8	14	24	5	11	8	11	305
Quarters positive with 3 bacterial species	1	2	3	5	1	2	6	6	4	2	9	1	1	6	2	2	3	0	3	1	5	1	1	4	4	4	79
Quarters positive only by one species NASM	18	17	15	4	22	4	14	15	16	10	8	6	9	7	10	7	12	14	13	8	15	14	17	7	1	4	287
Quarters positive only by two species NASM	24	4	10	23	8	6	17	11	11	10	12	1	5	12	4	5	12	7	15	7	13	23	5	7	2	3	257
Quarters positive only by three species NASM	1	2	3	5	1	2	6	6	3	1	8	1	1	6	1	2	3	0	3	1	5	1	1	3	2	1	69
**NASM and *S. aureus***
*Staphylococcus xylosus*	30	14	25	20	9	6	21	17	11	4	20	7	10	19	8	6	22	21	29	10	2	0	1	3	3	5	323
*Mammaliicoccus sciuri*	18	6	8	29	24	10	7	23	0	2	9	0	2	15	0	0	5	1	16	3	31	38	4	11	2	1	265
*Staphylooccus succinus*	17	2	1	5	1	1	17	1	5	1	2	0	0	0	0	3	6	2	0	3	3	0	0	2	1	0	73
*Staphylococcus equorum*	0	0	0	0	0	0	0	0	7	0	0	0	3	0	7	1	0	0	0	0	1	0	13	0	0	0	32
*Staphylococcus aureus*	0	0	0	0	0	0	0	0	0	2	0	0	0	0	0	2	7	3	2	3	0	0	0	11	2	0	32
*Mammaliicoccus vitulinus*	0	0	0	0	0	1	9	0	15	0	0	1	0	0	0	0	0	0	0	0	0	0	2	0	0	0	28
*Staphylococus chromogenes*	0	1	1	0	0	0	0	2	3	0	0	0	0	0	3	0	0	0	0	0	2	0	2	1	1	1	17
*Staphylococcus haemolyticus*	0	1	2	0	0	0	0	2	0	0	0	2	0	0	0	1	0	1	0	4	0	0	2	0	0	0	15
*Staphylococcus warneri*	0	0	0	0	0	0	0	0	0	11	0	0	0	0	0	2	0	0	0	0	0	0	0	0	0	0	13
Others*^1^*	1	0	0	3	0	0	1	3	0	3	2	0	0	0	0	3	0	1	0	2	0	0	2	0	0	0	21
*Bacillus cereus* group^2^	0	3	2	0	3	0	2	3	2	1	13	1	1	13	4	0	3	1	2	0	5	18	0	17	12	9	115
Others *Bacillus^3^*	0	2	0	0	0	1	0	1	0	0	2	0	0	1	0	0	0	0	1	0	0	0	0	0	1	0	9
*Acinetobacter* spp.*^4^*	0	0	4	0	0	0	0	0	1	2	1	0	1	0	0	1	3	0	0	0	0	2	1	1	4	2	23
*Aerococcus viridans*	0	0	0	0	0	3	1	0	0	2	0	0	4	0	0	4	1	2	0	0	2	0	2	0	0	0	21
*Arthrobacter* spp*.^5^*	0	0	0	0	0	0	0	1	1	0	3	1	4	1	4	0	0	0	0	0	0	0	0	2	0	7	24
*Enterococcus* spp.*^6^*	0	0	0	0	1	0	1	0	0	0	3	0	1	0	0	0	0	0	0	1	2	0	0	4	8	21	42
*Escherichia coli*	0	1	0	3	0	0	0	3	0	0	0	1	0	2	0	0	1	0	0	1	5	4	0	0	7	1	29
*Lactococcus* spp. *^7^*	0	0	0	0	0	0	0	0	0	1	2	0	4	2	0	10	6	0	0	0	0	0	0	0	0	0	25
*Streptococcus* spp.*^8^*	0	0	1	0	0	2	2	0	0	8	6	1	0	0	4	0	0	1	3	6	1	0	0	1	1	0	37
Others*^9^*	1	6	1	2	0	0	5	0	6	14	5	1	5	11	3	5	4	3	2	2	7	4	0	1	1	3	92
Not reliable identification	2	4	2	3	3	2	1	0	3	3	0	1	5	2	0	1	0	4	3	1	2	0	2	1	4	3	52
Total	1,288

Additional relevant information is provided, including the type of bedding used by the herds, the number of cows and the number of quarters. ^1^*Staphylococcus epidermidis* (2)*, Staphylococcus fleuretti* (2)*, Staphylococcus gallinarum* (5)*, Staphylococcus hyicus* (1)*, Staphylococcus lentus* (3)*, Staphylococcus simulans* (2)*, Staphylococcus spp.* (6). ^2^In this group are included *Bacillus cereus, Bacillus mycoides, Bacillus thuringiensis* (115). ^3^*Bacillus altitudinis* (1)*, Bacillus licheniformis* (2)*, Bacillus pumilus* (3)*, Bacillus simplex* (1)*, Bacillus subtilis* (1)*, Bacillus spp.* (1). ^4^*Acinetobacter towneri* (1)*, Acinetobacter calcoaceticus* (4)*, Acinetobacter guillouiae* (6)*, Acinetobacter johnsonii* (3)*, Acinetobacter lwoffii* (8)*, Acinetobacter spp.* (1). ^5^*Arthrobacter arilaitensis* (4)*, Arthrobacter gandavensis* (8)*, Arthrobacter histidinolovorans* (1)*, Arthrobacter ilicis* (1)*, Arthrobacter mysorens* (2)*, Arthrobacter nicotinovorans* (1)*, Arthrobacter protophormiae* (5)*, Arthrobacter spp.* (2). ^6^*Enterococcus faecium* (2)*, Enterococcus faecalis* (5)*, Enterococcus gallinarum* (1)*, Enterococcus saccharolyticus* (34). ^7^*Lactococcus garvieae* (16)*, Lactococcus lactis* (9). ^8^*Streptococcus agalactiae* (11)*, Streptococcus dysgalactiae* (1)*, Streptococcus parauberis* (7)*, Streptococcus uberis* (17)*, Streptococcus spp.* (1). ^9^*Aeromonas spp.* (3)*, Carnobacterium maltamaticum* (2)*, Citrobacter spp.* (5)*, Enterobacter cloacae* (3)*, Erwinia persicina* (1), *Klebsiella spp.* (10)*, Kluyvera spp.* (2)*, Kocuria spp.* (2)*, Lelliotta amnigena* (2)*, Lysinibacillus sphaericus* (3)*, Microbacterium arboriscens* (1). *Micrococcus luteus* (1)*, Moraxella spp.* (1)*, Neisseria macacae* (1)*, Paenibacillus spp.* (1)*, Pantoea agglomerans* (8)*, Proteus spp.* (9)*, Providencia rettgeri* (2)*, Pseudomonas spp.* (21)*, Rahnella aquatilis* (1)*, Raoultella spp.* (4)*, Serratia marcescens* (5)*, Trueperella pyogenes* (2)*, Vagococcus fluvialis* (1)*, Vibrio cincinnatiensis* (1).

### 2.2. Analysis of somatic cell counts

Somatic cell counts (SCC) in individual quarter milk samples (identical to those used for bacteriological analyses, see below) were used to differentiate between healthy quarters and those with subclinical mastitis (cows and quarters with clinical forms of mastitis were strictly excluded from the study). According to the International Dairy Federation (IDF), a quarter was considered healthy if SCC were < 200,000 cells/mL, independent on number and stage of lactation (International Dairy Federation, 2022). Values above were considered as a quarter with subclinical mastitis. Total SCC were analyzed in frozen milk samples using the recently published flow cytometry method by Widmer et al. (2022). For analysis, the samples were defrosted at room temperature and gently mixed by inversion. The impact of freezing on SCC using this method was tested with 120 raw milk samples and the average decrease in cell numbers was 6.3%.

### 2.3. Bacteriological analyses and identification

Bacteriological analyses were performed following the *Laboratory Handbook on Bovine Mastitis* of the National Mastitis Council [National Mastitis Council (NMC), 2017]. In brief, 10 μL of each milk sample was streaked out on sheep blood agar (BA) plates (Biomèrieux Suisse SA, Geneva, Switzerland) and bacterial colonies obtained after 24 and 48 h of aerobic incubation at 37°C were evaluated based on their morphology. Samples were considered “contaminated” and not included in the study, if more than 3 morphologically different bacterial colonies could be identified (Wyder et al., 2011). Representatives of each colony type were selected for bacterial identification using MALDI-TOF MS according to the protocols of the manufacturer (Bruker Daltonics GmbH, Bremen, Germany). Analysis was performed by a Microflex LT MALDI-TOF instrument using the MALDI Biotyper (MBT) Compass Library 7,311 (both Bruker Daltonics GmbH). Isolates with a score ≥ 2.2 were identified at the species level. All distinct isolates were conserved in skim milk at-20°C for subsequent analyses.

### 2.4. Selection of isolated bacterial species for resistome analysis

By analyzing all morphologically distinct colonies for each milk sample by MALDI TOF MS, a total of 1,288 isolates were obtained. To reduce the number of isolates and to restrict it to the most relevant bacteria for each herd and sampling, the following selection procedure was performed: (1) For each BA plate, all morphologically different colonies with an abundance ≥5 were taken, identified, and the corresponding bacteria registered in a frequency table. The table was then sorted in descending order according to the observed frequencies. Starting from top, bacteria were selected until the sum of their frequencies resulted in ≥85% of the total frequency. (2) For each of these selected, relevant bacteria, 5 isolates (if available) were then randomly chosen resulting overall in 350 isolates that were later subjected to phenotypic AMR testing and bioinformatic ARGs analysis after whole genome sequencing (WGS).

### 2.5. Phenotypic antimicrobial susceptibility testing

Minimum inhibitory concentrations (MIC) were determined for all 350 isolates using different antibiotics panels that accounted for the respective characteristics of the bacterial species. All tests were performed according to the manufacturer’s instructions of the Microscan System (Beckman Culture Microbiology, West Sacramento, CA). For details see the Supplementary Methods Section in the Supplementary materials.

### 2.6. Whole genome sequencing

Details concerning the extraction of genomic DNA for short and long read sequencing platforms to then first *de novo* assemble, polish and annotate complete genomes of type strains (15 of 23 that lacked a complete genome at NCBI) are described in detail in the Supplementary Methods, along with the respective strategies to assemble the reads even from highly repeat-rich and complex strains (Schmid et al., 2018). The complete type strain genomes served as a basis for reference-based assembly for the 350 WGS sequenced isolates (Illumina HiSeq platform) (Supplementary Tables S1–S4).

The 350 isolates were plated on sheep blood agar (BA) plates (Biomèrieux Suisse SA, Geneva, Switzerland) and incubated aerobically at 37°C for 18 h (h). Two to four single colonies were picked, resuspended in 5 mL BHI (Brain Heart Infusion Broth, Merck KGaA, Darmstadt, Germany) and incubated under the same conditions for 18 h. Subsequently, 200 μL of the pre-cultures were added to 100 mL of fresh BHI and incubated aerobically at 37°C for 18 h under constant shaking, before 50 mL were collected and centrifuged (18,000 × *g* for 5 min at 4°C). The supernatant was discarded, the pellet resuspended in 600 μL of buffer A1 (NucleoSpin^®^ 8 Plasmid kit, Macherey-Nagel AG, Oensingen, Switzerland) and DNA isolated according to the manufacturer’s protocol. The total amount and quality of DNA were evaluated by spectroscopy assessing the OD_260_/OD_280_ ratio (QuickDrop; Molecular Devices, San Jose, CA) and a quantitative analysis (Qubit assay; Thermo Fisher Scientific, USA).

### 2.7. WGS and assembly

DNA samples (*n* = 350) were sequenced by Eurofins Genomics GmbH (Ebersberg, Germany) on the HiSeq sequencing platform (Illumina, San Diego, CA). The reads were first assembled using the complete genome of the type strain of the corresponding species as reference (Supplementary files *Illumina and long reads sequencing (PacBio/ONT) of type strains*) using SeqMan NGen 16 software (default settings) from the DNASTAR Lasergene 16 software package (DNASTAR Inc., Madison, WI). The unassembled reads were *de novo* assembled with the *de novo* task (with the parameters deactivated ‘repeat handling’ option, minimum read overlap match of 93%, and contigs longer than 1,000 nucleotides). The assembled chromosomes and contigs were next annotated with the RAST pipeline.^2^

### 2.8. *In silico* identification of antimicrobial resistance genes

The assembled genomes (chromosomes and contigs) of the 350 isolates were analyzed for the presence of antimicrobial resistance genes (ARGs) using three different approaches: an in-house manually curated fasta database for AMR genes of *Staphylococcus* spp. together with CM software (Supplementary file), and two online software tools, i.e., ResFinder^3^ (Bortolaia et al., 2020) and Resistance Gene Identifier RGI^4^ (Alcock et al., 2020, Comprehensive Antibiotic Resistance Database, CARD, 2019). An additional manual check to evaluate the functionality of the ARGs was performed (Clone Manager v9.51; CM9; Sci Ed Software, Westmister, CO) by comparing the reference gene to that of the respective isolate. To leverage the benefits of curated databases (SIB, Swiss Institute of Bioinformatics Members, 2016) all ARGs were compared with CARD to establish an association between the genes and the respective antimicrobial compounds against which their encoded products act.

### 2.9. Analysis of mutations in the *mecA1* gene of *Mammaliicoccus sciuri* isolates

For 20 of the 83 *Mammaliicoccus sciuri* isolates, the promoter region of the *mecA1* genes was analyzed manually using Clone Manager v9.51 software (CM9Sci Ed Software, Westmister, CO). A sample was considered positive, when a *mecA1* promoter mutation was detected at position-10 as reported in previous studies (Wu et al., 2001; Frey et al., 2013).

### 2.10. *In silico* analysis of plasmids

The assembled chromosomes and contigs of all bacterial species were analyzed for the presence of plasmids using the PlasmidFinder^5^ software (Carattoli et al., 2014); if positive, they were further analyzed using the curated database PLSDB^6^ (Schmartz et al., 2022).

### 2.11. Additional analysis of tetracycline AMR

All reads from isolates that exhibited matches to the *tetK* gene were mapped against the *tetK* reference gene (1,380 bp; NCBI, GenBank: S67449.1) downloaded from the CARD database using SeqMan NGen 16 (default settings). Alignments were manually checked using the Clone Manager 9.51 software (CM9; Sci Ed Software, Westmister, CO). Additionally, plasmid SPADES and Unicycler were used to circularize the plasmids of four randomly selected isolates that carried the *tetK* gene. Subsequently, all reads of isolates positive for the *tetK* gene were assembled with the closed plasmids as references and compared with Clone Manager 9.51.

### 2.12. Statistics

Descriptive statistics for bacterial prevalence at the cow and quarter level were performed using Microsoft Excel 2016 (Microsoft Corporation). Additionally, descriptive statistical analyses were achieved to evaluate the percentage of healthy and inflamed (subclinical mastitis) quarters that showed a monoinfection with *S. xylosus* and *M. sciuri*, respectively.

A Fisher’s exact test was used to test if there was non-random association between the bacteria and the distribution in different herds. Additionally, a generalized version of the Fisher’s exact test for k x m tables was performed to test the association between herds and resistance to the main antibiotics involved (azithromycin, clindamycin, oxacillin, penicillin, and tetracycline). The same test was further performed to evaluate a possible association between milk sampling and lactation stage of the sampled cows.

To assess the impact of the stage of lactation on the intramammary presence of *S. xylosus* and *M. sciuri*, for each bacterium a loglinear model was computed, both at the cow and at the quarter level. The models included the factors bacterium, stage of lactation, farms, and their interactions. The very same procedure was used to assess the impact of the lactation number on intramammary *S. xylosus* and *M. sciuri*. For both *S. xylosus* and *M. sciuri*, their overall presence was evaluated including mono-and co-cultures with other bacteria.

For all statistical analyses except stated the Systat 13.1 software (Systat Software, San Jose, CA) was used. A value of *p* < 0.05 was considered significant.

### 2.13. Data availability

The complete genomes sequences (and sequences of the plasmids) for 15 type strains that were *de novo* assembled and used as reference genomes are available under Bioproject PRJNA936091.^7^ The raw Illumina reads obtained from WGS of 350 isolates are publicly available from NCBI GenBank under Bioproject PRJNA859642.^8^ Our manually curated database of *Staphylococcus* spp. ARGs is released as Supplementary Word file.

## 3. Results

**Figure 1:**
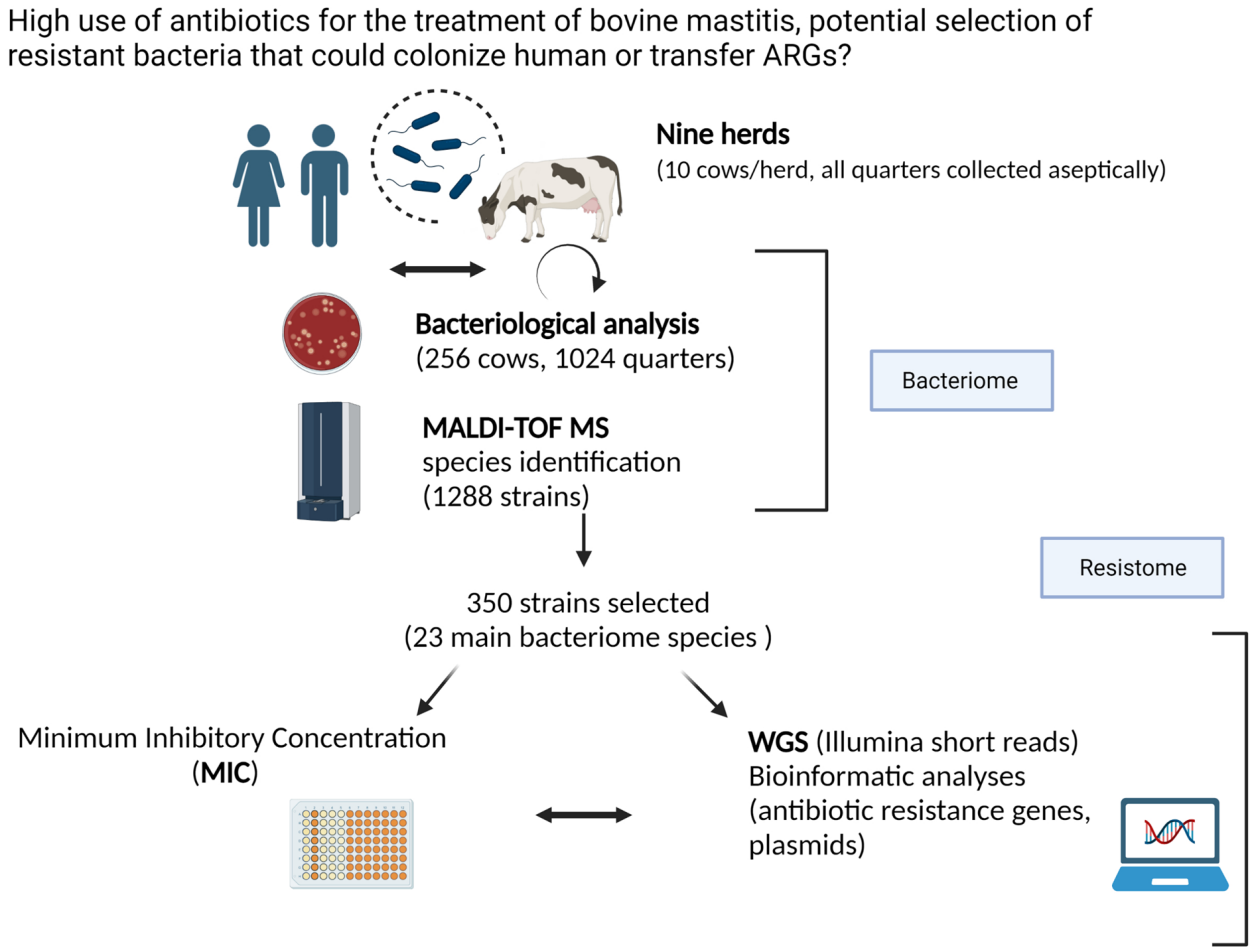
Graphical abstract representing an overview of the on-farm sampling (nine dairy cow herds from the Canton Tessin) and the experimental laboratory procedure to describe the bacteriome and resistome of healthy cows (created in Biorender.com). Confirmation of Publication and Licensing Rights.

The results are organized along the main two themes of the present study, i.e., the intramammary bacteriome and resistome, respectively (Figure 1).

### 3.1. Composition of the intramammary bacteriome

**Figure 2:**
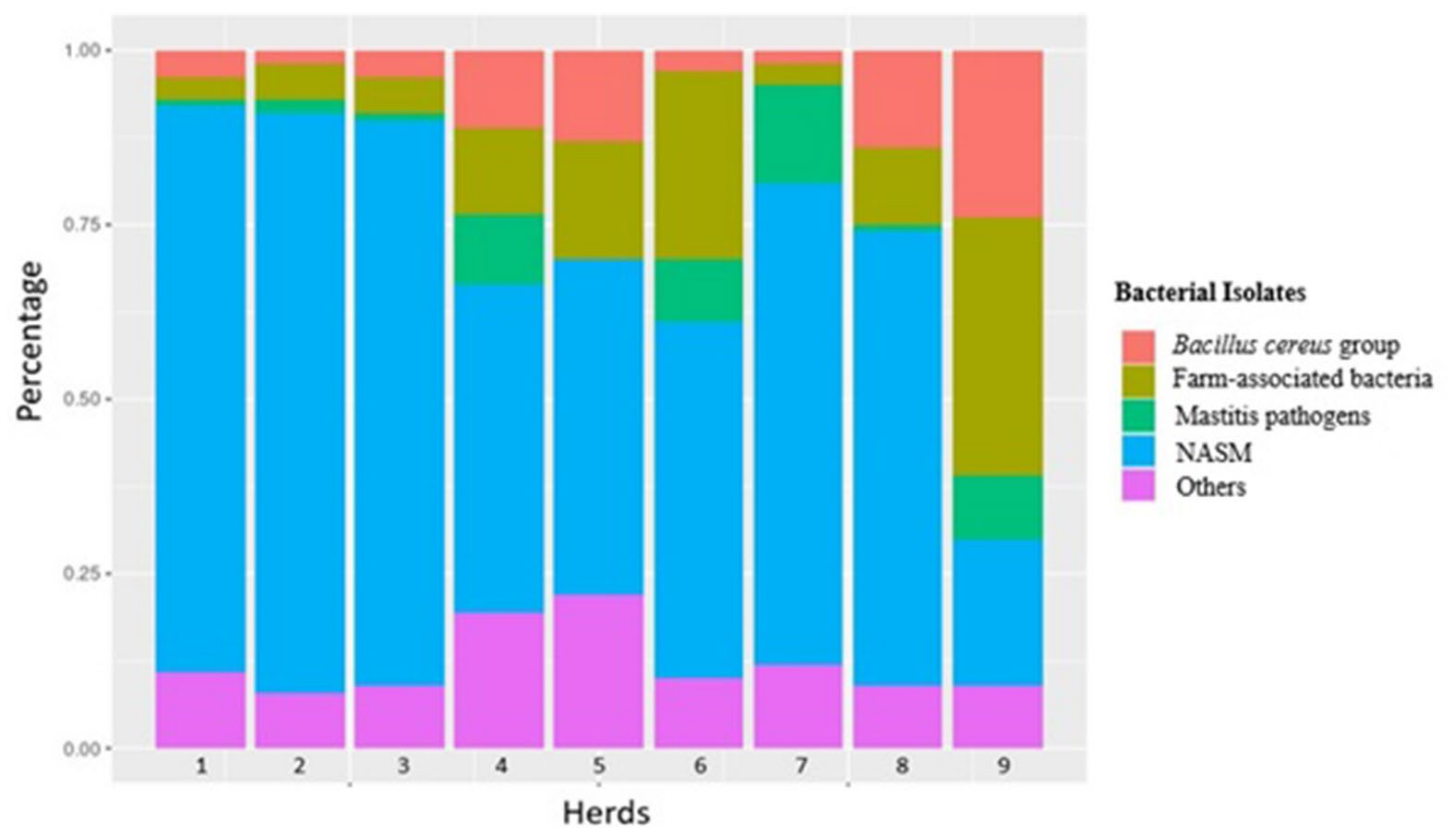
Distribution of the main bacterial groups identified in the nine different herds over the three sampled time points.

The composition of the intramammary bacteriome and distribution of individual species of each single herd at the three sampling time points is summarized in Table 1 and Figure 2. An additional figure showing the distribution of the different bacteria during sampling time (T0, T1, T2) is included in the Supplementary material (Supplementary Figure S1). Overall, a total of 1,024 milk samples (from 256 cows) collected aseptically from each quarter were analyzed. For each herd and time point, 10 randomly selected healthy cows were sampled (unless otherwise stated, see Materials and methods). The average age of the cows ranged between 4.4 to 8.9 years. 78.4% (*n* = 200) of the cows were multiparous, while 21.6% (*n* = 55) were primiparous. No data was available for two cows. Another parameter collected was the lactation stage; 108 cows were in late lactation, 76 in mid, and 70 in early lactation. These data are listed in a table integrated in the Supplementary material (Supplementary Table S5).

To assess the impact of the stage of lactation on the intramammary presence of *S. xylosus* and *M. sciuri*, the most frequently observed bacteria in this study, a loglinear model was computed for each bacterium, both at the cow and at the quarter level. The models included the factors bacterium, stage of lactation, farms, and their interactions. The variable sampling time (T0, T1, T2) was omitted from the model as a strong association was observed between this variable and stage of lactation (*p* < 0.001). Indeed, at T1 most cows were in mid (29%) and late lactation (62%). The 4 loglinear models were also used to assess the impact of the lactation number on intramammary *S. xylosus* and *M. sciuri*. The results showed that at the cow level the intramammary presence of *S. xylosus* was independent on the stage of lactation (*p* = 0.840) but dependent on the farm (*p* < 0.001). Different was the situation at the quarter level where a dependency for stage of lactation was observed (*p* = 0.004). Regarding *M. sciuri*, intramammary presence at the cow level was farm dependent (*p* < 0.001), but was independent on the stage of lactation (*p* = 0.515). At the quarter level, intramammary *M. sciuri* positivity was farm (*p* < 0.001) and lactation stage dependent (*p* = 0.001). Considering lactation number, it did not affect intramammary presence of *S. xylosus* at the cow level (*p* = 0.652), whereas an effect was observed at the quarter level (*p* = 0.015). For *M. sciuri*, the loglinear model showed no dependency as well as at the cow (*p* = 0.131) as at the quarter level (*p* = 0.076).

The SCC data demonstrated overall a high number of quarters (84.5%) with SCC below of 200,000 cells/mL and were, therefore, considered healthy. The remaining 15.5% were considered as quarters with subclinical mastitis. For *S. xylosus* explicitly, 81% of the quarters showed values below 200,000 cells/mL, with a median SCC of 36,740 cells/mL, while for *M. sciuri*, 92% of the quarters were considered healthy (SCC < 200,000 cells/mL) with a median SCC of 18,920 cells/mL.

250 of the 256 cows were bacteriologically positive for at least one quarter (98%): nineteen cows were positive for one quarter (7%), 28 cows for 2 quarters (11%), and 64 (26%) and 139 (56%) for 3 or 4 quarters, respectively. At the quarter level, 824 of the overall 1,024 milk samples were bacteriologically positive (80%). The prevalence of positive quarters in the different herds ranged from 30 to 100% (Table 1). The median values for the sampling time point T0, T1, and T2 were 87, 89, and 63%, respectively. Overall, 440 quarters were colonized by one bacterial species (53%), 305 by 2 bacterial species (37%), and 79 (10%) by 3 different species. In 613 quarters (74%) *Staphylococcus* spp. or *Mammaliicoccus* spp. were detected: 287 quarters were positive for one species (47%), 257 (42%) for 2 different species, and 69 (11%) for three different species (Table 1). The percentage of bacteriologically positive quarters differed between the herds. For the one farm that used extracted and compressed manure particles, 5 of 31 quarters (16%) were positive for NASM in the second sampling. For the other 8 farms that used straw and sawdust for bedding, the median prevalence for NASM at the quarter level was 79%.

*S. xylosus* was the most frequently identified isolate; it was detected in all herds and sampling time points except for herd 8 at T2. In herds 8 and 9, a much lower prevalence compared to the other herds was recorded (Table 1). In herds 1, 5, and 7, *S. xylosus* represented the main species in all three samplings, while in herd 2, *M. sciuri* was predominantly detected. For the other herds, different patterns were found. In herd 3, NASM were the main bacteria, mainly represented by *S. xylosus*, *M. sciuri* and *Mammaliicoccus vitulinus* with differences within the samples. In herd 4, the first sampling was colonized by *Staphylococcus warneri*, while in the second and third sampling, *S. xylosus* was mainly identified. Farm number 6 was sampled only twice. The first sampling included mainly *Lactococcus* spp. and the second was mainly composed of *S. xylosus*. At farm number 8, the first 2 sampling included *S. xylosus*, while in the third *Staphylococcus equorum* was mainly detected. Cows from herd 9 displayed a completely different pattern of isolates. In the first and second sampling mainly bacteria from the *Bacillus cereus* group were isolated. In the third sampling *Enterococcus* spp. was predominantly detected. To evaluate a non-random association between the herds and groups of isolated bacteria (*S. xylosus*, *M. sciuri*, *Bacillus cereus* group, farm-associated bacterial, and mastitis pathogens), a Fisher exact test was performed. The Exact test uncovered a statistically significant association (*p* < 0.05) between the herds and the bacteria isolated (*S. xylosus*, *M. sciuri*, *Bacillus cereus* group, farm-associated bacterial, mastitis pathogens) implying a distinct distribution of the groups in the different herds.

**Figure 3:**
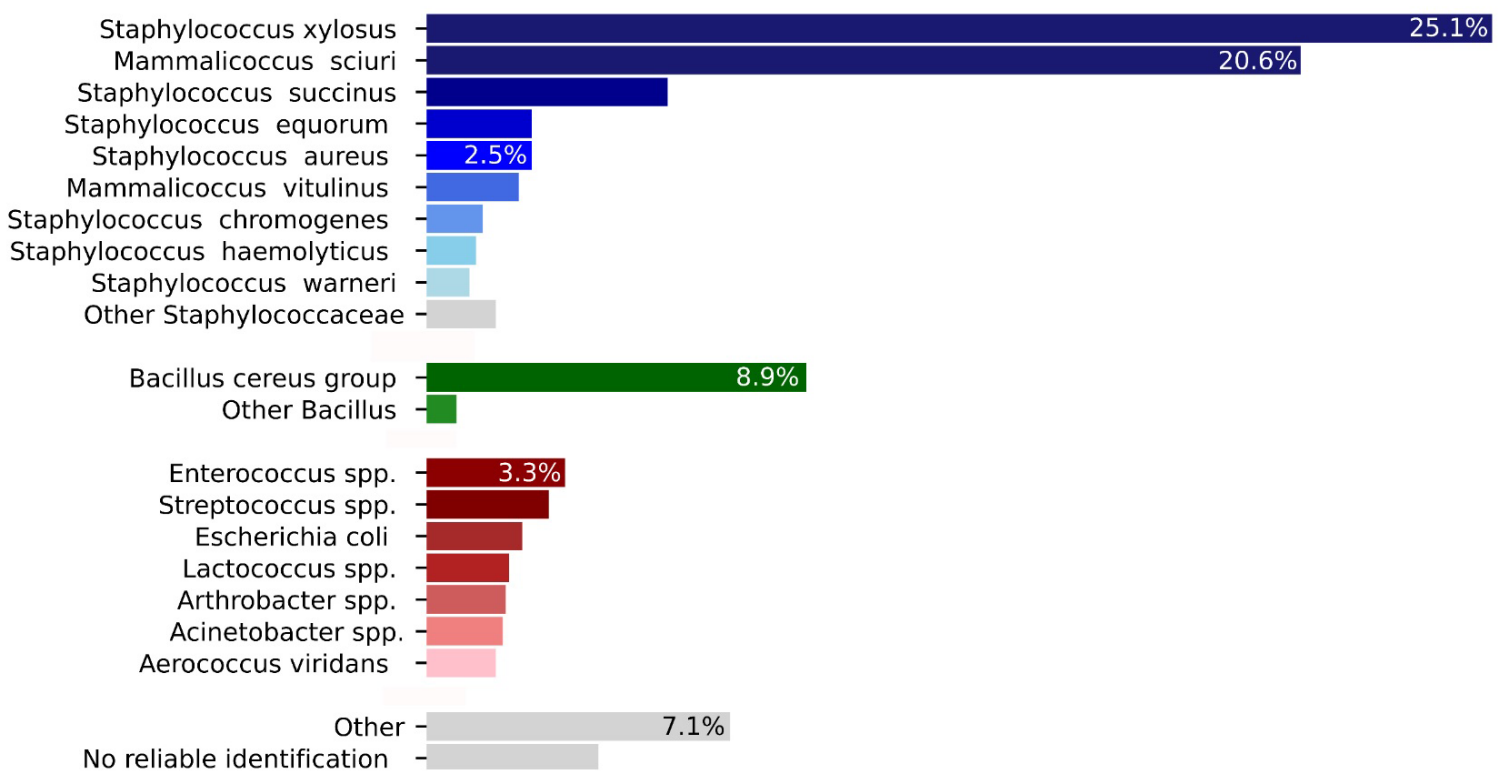
Graphical summary of the distribution of the 1,288 bacterial isolates from Table 1 included in the main text (total number and percent). The bacterial species are divided accordingly with the main table.

The 1,288 isolates that were isolated and identified, belonged to 104 different bacterial species (Table 1). The intramammary bacteriome compositions displayed a herd-specificity with an overall very high prevalence for NASM (Figure 3). The percentage of bacteria known to cause mastitis [*S. aureus* (2.5%), *S. uberis* (1.3%), and *Streptococcus agalactiae* (0.85%)] was lower in relation to the other categories listed above. *S. xylosus* (323 isolates) and *M. sciuri* (265 isolates) were by far the most prevalent detected bacteria in milk (for a total of 46%, 588 isolates) (distribution of the different NASM explained in the Supplementary Figure S2), followed by the *Bacillus cereus* group (9%). *S succinus* (6%) and *Enterococcus saccharolyticus* (3%), and *Escherichia coli* (2.2%) represented further potentially pathogenic bacteria. 52 isolates (4%) could not be identified by MALDI-TOF MS typing. Figure 3 shows a graphical distribution of all bacterial isolates.

### 3.2. Intramammary resistome

Among all isolates, 350 isolates were selected to be analyzed in more detail with respect to their genomic sequence and phenotypical antimicrobial resistance profile. To study the AMR genes and the phenotypical resistome, we chose a subset of isolates that represented the most abundant species as described in the Materials and Methods section. The data which species were identified in the study and the corresponding number of isolates are listed in Table 2.

**Table 2:** Bacteria selected for Illumina-based WGS and phenotypic antibiotic analysis.

Bacterial species isolates	Milk T0	Milk T1	Milk T2	Sum per species
*Staphylococcus xylosus^#^*	35	38	28	101
*Mammaliicoccus sciuri^#^*	27	40	16	83
Staphylococcus succinus*	13	4	5	22
Staphylococcus equorum*	3	0	13	16
*Staphylococcus aureus^#^*	4	0	5	9
Mammaliicoccus vitulinus*	5	0	4	9
Staphylococcus warneri*	5	0	0	5
Staphylococcus haemolyticus*	2	0	1	3
Staphylococcus chromogenes*	0	0	3	3
Acinetobacter lwoffii*	2	0	0	2
Aerococcus viridans*	6	0	3	9
Arthrobacter gandavensis*	2	0	0	2
*Bacillus cereus group^#^*	10	25	0	35
Enterococcus faecalis*	0	1	0	1
Enterococcus faecium*	0	2	0	2
Enterococcus saccharolyticus*	3	3	0	6
*Escherichia coli ^#^*	4	9	0	13
Lactococcus garvieae*	5	2	0	7
Lactococcus lactis*	4	3	0	7
*Streptococcus agalactiae ^#^*	6	5	0	11
*Streptococcus uberis ^#^*	0	0	4	4
Total number				350

The respective frequency of the bacterial species isolated at three different sampling time points is listed. Hash-tag symbols denote species for which a complete type strain genome was available at the NCBI. Asterisks denote species for whose type strain a *de novo* assembly was carried out combining data from 3^rd^ generation long read sequencing technologies (PacBio and/or ONT) and the Illumina short read platform (see Supplementary Table S2).

#### 3.2.1. Phenotypic AMR

**Figure 4:**
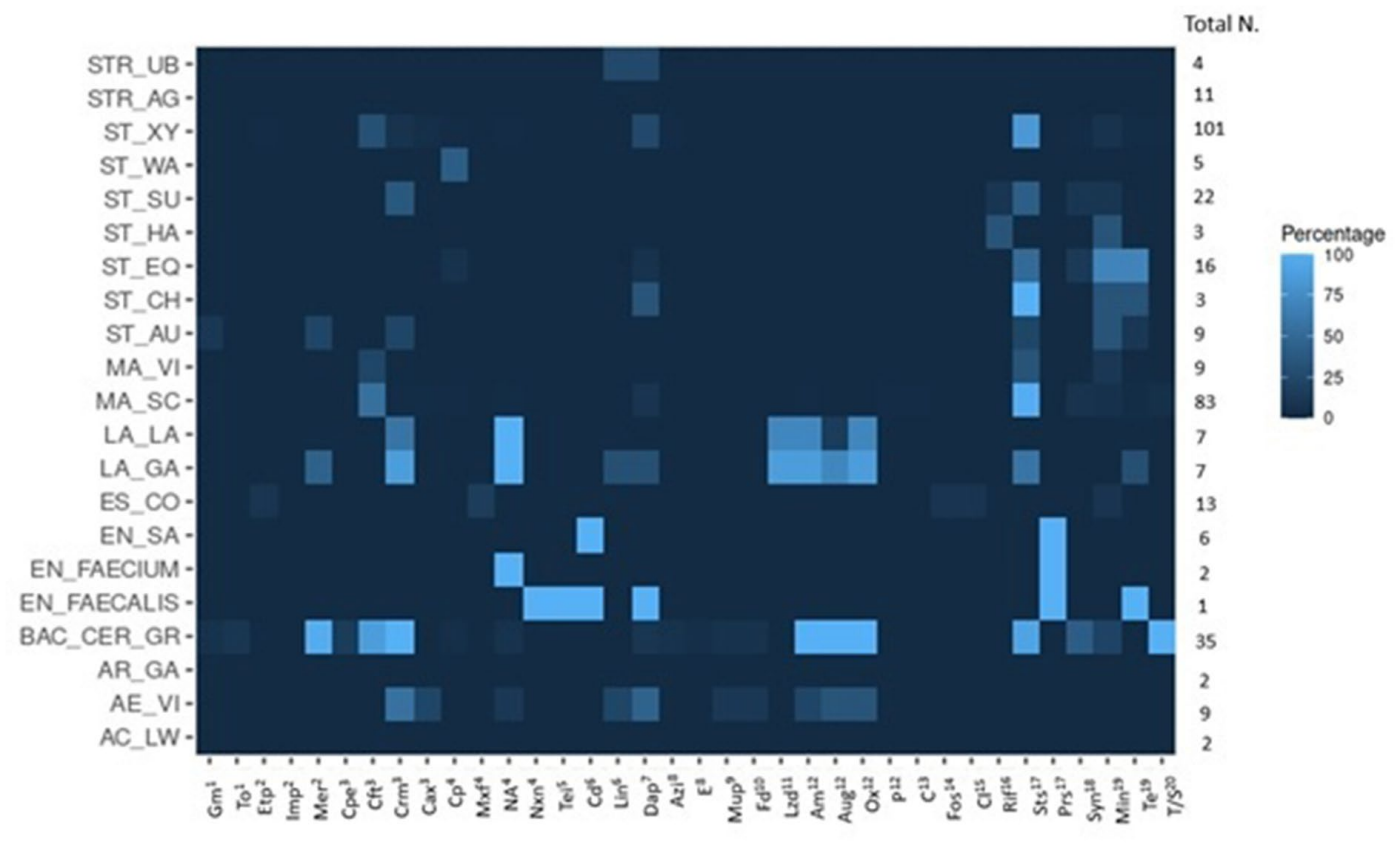
Graphical heatmap showing the results displayed in the Table 4. The different color scale represents the percentage of isolates resistant to the different antibiotics checked.

Clindamycin and oxacillin resistance were most often observed among the 350 isolates with a total of 227 isolates (65%) resistant to clindamycin and 105 isolates (30%) to oxacillin (Table 3). The main resistant isolates were assigned to the NASM, and *Bacillus cereus* families. In total, 18% of the isolates were sensitive to all tested antibiotics, 25% of the isolates (88) were resistant to one and 28% (97) to two different antibiotics. Fifteen percent of the isolates (54) displayed AMR to more than four antibiotics. These isolates mainly belonged to the multi-resistant *Bacillus cereus* group. The distribution of the antibiotic resistances and percentage of the phenotypic resistances are listed in Tables 3, 4 (Graphical representation heatmaps Figure 4). The data of the phenotypic results for all species analyzed are listed in Supplementary Table S7.

**Table 3:** Distribution of the overall number of phenotypic antibiotic resistances exhibited by the different bacterial isolates analyzed in this study.

Bacteria isolates	No R	R 1 AA	R 2 AA	R 3 AA	R 4 AA	R > 4 AA
*Staphylococcus xylosus*	11	**37**	34	15	4	
*Mammaliicoccus sciuri*	4	27	**35**	14	1	2
*Staphylococcus succinus*	7	7	**8**			
*Staphylococcus equorum*	2	2	5	**6**	1	
*Staphylococcus aureus*	**4**	1	1	1		2
*Mammaliicoccus vitulinus*	**6**	3				
*Staphylococcus warneri*	**3**	2				
*Staphylococcus haemolyticus*	1	**2**				
*Staphylococcus chromogenes*	**2**				1	
*Acinetobacter lwoffii*	**2**					
*Aerococcus viridans*	1	1	2	1		**4**
*Arthrobacter gandavensis*	**2**					
*Bacillus cereus* group					1	**34**
*Enterococcus faecalis*						**1**
*Enterococcus faecium*			**2**			
*Enterococcus saccharolyticus*			**6**			
*Escherichia coli*	**10**	2				1
*Lactococcus garvieae*			1			**6**
*Lactococcus lactis*		2				**5**
*Streptococcus agalactiae*	**11**					
*Streptococcus uberis*	**3**		1			
Total	69	86	95	37	8	55

The table shows the number of resistances observed for the isolates, which could range from resistance to one antibiotic (AB) up to resistance to over four ABs. R, resistance; AB, antibiotics. The meaning of the bold values represent the majority of the samples resistance to the antibiotics.

**Table 4:** Percentage of phenotypic resistance observed against different antibiotics (based on the MIC assays).

Bacteria isolates species	Gm^1^	To^1^	Etp^2^	Imp^2^	Mer^2^	Cpe^3^	Cft^3^	Crm^3^	Cax^3^	Cp^4^	Mxf^4^	NA^4^	Nxn^4^	Tei^5^	Cd^6^	Lin^6^	Dap^7^	Azi^8^	E^8^	Mup^9^	Fd^10^	Lzd^11^	Am^12^	Aug^12^	Ox^12^	P^12^	C^13^	Fos^14^	Cl^15^	Rif^16^	Sts^17^	Prs^17^	Syn^18^	Min^19^	Te^19^	T/S^20^	Total N. isolates
ST_XY			1												83		1	7	2	2	1				31	7	3	1		1					25		101
MA_SC	1						1			1	1				98		7	5	2	4					54	1	2	1		2					8		83
ST_SU														9	41		9	9								36											22
ST_EQ															50		13	69	69									6							6		16
ST_AU	11														22			33	11				22			22											9
MA_VI															33			11							22												9
ST_WA																												40									5
ST_HA														33				33																			3
ST_CH															100			33	33																33		3
AC_LW																																					2
AE_VI					11	33	22	33																		55	22			11				22	44	11	9
AR_GA																																					2
BAC_CER_GR	6	9	6	3	6	100	100	100							91		40	20		100			97	14	86	100		3		6					9	6	35
EN_FAECALIS																100			100												100	100	100		100		1
EN_FAECIUM																100														100							2
EN_SA																100																	100				6
ES_CO												8	8					8			8								15								13
LA_GA						71	86	86	86						57				29				43			86				100				29	29		7
LA_LA						14	71	71	71																	57				100							7
STR_AG																																					11
STR_UB																																		25	25		4
Total isolates	350

The table displays the percentage of the resistant isolates, i.e., compared to the total number of isolates per species. The antibiotics were arranged according to their classification into different classes (superscript numbers 1 to 20; see legend). Bacterial species listed: ST_XY: *Staphylococcus xylosus*, MA_SC: *Mammaliicoccus sciuri*, ST_SU: *Staphylococcus succinus*, ST_EQ: *Staphylococcus equorum*, ST_AU: *Staphylococcus aureus*, MA_VI: *Mammaliicoccus vitulinus*, ST_WA: *Staphylococcus warneri*, ST_HA: *Staphylococcus haemolyticus*, ST_CH: *Staphylococcus chromogenes*, AC_LW: *Acinetobacter lwoffii*, AE_VI: *Aerococcus viridans*, AR_GA: *Arthrobacter gandavensis*, BAC_CER_GR: *Bacillus cereus group*, EN_FAECALIS: *Enterococccus faecalis*, EN_FAECIUM: *Enterococcus faecium*, EN_SA: *Enterococcus saccharolyticus*, ES_CO: *Escherichia coli*, LA_GA: *Lactococcus garvieae*, STR_AG: *Streptococcus agalactiae*, STR_UB: *Streptococcus uberis*. Classification of different antibiotics: 1 Aminoglycoside (Gm: gentamycin, To: tobramycin), 2 Carbapenem (Etp: ertapenem, Imp: imipenem), 3 Cephalosporins (Cpe: cefepime, Cft: cefotaxime, Crm: cefuroxime, Cax: ceftriaxone) 4 Fluorochinolones (Cp: ciprofloxacin, Mxf: moxifloxacin, NA: nalidixic acid, Nxn: norfloxacin) 5 Glycopeptides (Tei: teicoplanin), 6 Lincosamides (Cd: clindamycin, Lin: lincomycin) 7 Lipopeptides (Dap: daptomycin), 8 Macrolides (Azi: azithromycin, E: erythromycin), 9 Mupirocins (Mup: mupirocin), 10 Nitrofurantoin (Fd: nitrofurantoin), 11 Oxazolidinones (Lzd: linezolid), 12 Penicillin (Am: ampicillin, Aug: amoxacillin/ K clavulanate, Ox: oxacillin, P: penicillin) 13 Phenicols (C: chloramphenicol), 14 Fosfomycins, (Fos: fosfomycin)15 Polymyxins (CI: colistin), 16 Rifampicins (Rif: rifampin), 17 Streptogramins (Sts: streptomycin, Prs: pristamycin), 18 Synercyd (Syn), 19 Tetracyclines (Min: minocycline, Te: tetracycline), 20 Trimethoprim/sulfamethoxazole (T/S).

##### 3.2.1.1. *Staphylococcus* spp.

For 17 of the 31 antibiotics tested in this study, we identified at least one isolate that was resistant. Eighteen percent of the *Staphylococcus* spp. isolates were sensitive to all tested antibiotics, 34% were resistant to one, 30% to two antibiotics, and the remaining 18% showed multiple resistances for up to six antibiotics. Resistance to clindamycin (116, 73%), oxacillin (31, 20%), tetracycline (26, 16%), azithromycin (25, 16%), and penicillin (14, 9%) were detected most often. In detail, *S. xylosus* were phenotypically resistant to clindamycin in 83% of the isolates, oxacillin (31%), and tetracycline (25%). Fifteen percent of the *S. xylosus* isolates were multi-drug resistant (MDR) for more than 3 classes of antibiotics. For *Staphylococcus succinus* 41% of the isolates showed resistance to clindamycin and 36% to penicillin. *Staphylcoccus equorum* isolates mainly exhibited resistance to azithromycin and erythromycin (macrolides) (69%), and 50% of the isolates were resistant to clindamycin. *S. warneri* isolates only showed resistance to fosfomycin. *Staphylococcus haemolyticus* isolates were resistant to azithromycin and teicoplanin. All *S. chromogenes* isolates showed resistance to clindamycin; interestingly, one isolate was also resistant to macrolides (azithromycin and erythromycin) and tetracycline. In summary, in *Staphylococcus* spp., a high resistance rate to clindamycin, oxacillin, tetracycline and azithromycin was detected.

##### 3.2.1.2. *Mammaliicoccus* spp.

*M. sciuri* and *M. vitulinus* were mainly resistant to clindamycin (87%) and oxacillin (52%). Of 83 *M. sciuri* isolates, 91% were resistant to clindamycin, 54% to oxacillin, and 8% to tetracycline. Twenty-nine isolates presented a resistance to both clindamycin and oxacillin (35%), and 32% were limited to clindamycin. Additional AMRs to daptomycin (6 isolates: 7%), azithromycin (4 isolates: 5%) and mupirocin (3 isolates: 4%) were observed. 5% of the *Mammaliicoccus* spp. isolates were sensitive to all antibiotics analyzed in this study. For *M. vitulinus*, five out of nine isolates were sensitive to all antibiotics (56%), one isolate was resistant to azithromycin and two displayed a resistance to oxacillin.

The Exact test revealed a highly significant association between the NASM species (*S. xylosus, M. sciuri, S. equorum, S. succinus*) and the five main antibiotic resistances to azithromycin, clindamycin, oxacillin, penicillin, and tetracycline; for all species analyzed (*p* < 0.001). For *S. xylosus* and *M. sciuri*, the two most prominent species, the Exact test showed only a significant association for *S. xylosus* between the herds and tetracycline resistance (*p* < 0.001). A resistance to tetracycline was detected in six out of the nine herds.

##### 3.2.1.3. *Bacillus cereus* group

The isolates of the *Bacillus cereus* group were mostly multi-drug resistant isolates. All isolates were resistant to more than six antibiotics, except one isolate, which was only resistant to four antibiotics. All 35 isolates exhibited resistances to cefepime, cefotaxime, cefuroxime, and penicillin; additionally, 34 out of 35 were resistant to ampicillin. These results indicated a high resistance to β-lactam antibiotics. Only three isolates were resistant to tetracycline (9%), but a high proportion was resistant to clindamycin (32 isolates, 91%).

##### 3.2.1.4. Farm associated bacteria

Further research on antibiotic resistance was performed with bacterial isolates from *Acinetobacter lwoffii*, *Aerococcus viridans*, *Arthrobacter gandavensis*, *Enterococcus* spp., *E. coli*, *Lactococcus* spp., and *Streptococcus* spp.

For *Acinetobacter lwoffii* and *Arthrobacter gandavensis*, no resistance to any of the tested antibiotics was detected. Regarding *Aerococcus viridans*, the breakpoints were not defined for all tested antibiotics. Based on the EUCAST guidelines for *Aerococcus* spp., only the resistance to amoxicillin/K clavulanate, ampicillin, levofloxacin, meropenem, penicillin, rifampin, and vancomycin could be defined. Based on this observation, we isolated one isolate resistant to meropenem and another one to rifampin. Additionally, five isolates from two different farms were resistant to penicillin (55%). We identified additional resistances to cephalosporins, chloramphenicol, tetracycline (44%) and trimethoprim/sulfamethoxazole. All bacteria of the *Lactococcus* spp. displayed resistance to rifampin and to different categories of β-lactam antibiotics.

All *Enterococcus* species were resistant to lincomycin. Two isolates of *Enterococcus faecium* were additionally resistant to rifampin, while six isolates of *Enterococcus saccharolyticus* were also resistant to synercid. The isolates of *Enterococcus faecalis* displayed multi-resistances to erythromycin, gentamicin, pristinamycin, streptomycin, synercid, and tetracycline.

Ten out of 13 analyzed *E. coli* isolates were sensitive to all the antibiotics tested; two isolates were resistant to colistin and one isolate was resistant to aztreonam, nalidixic acid, nitrofurantoin, and norfloxacin.

##### 3.2.1.5. Mastitis pathogens

Among the nine *S. aureus* isolates, two isolates showed only resistance to azithromycin and clindamycin, one isolate to azithromycin and the β-lactams ampicillin and penicillin. Additionally, one isolate was MDR. Considering the 15 *Streptococcus* spp. (11 *S. agalactiae* and four *S. uberis*), 14 were sensitive to all antibiotics (93.3%). One isolate of *S. uberis* was exclusively resistant to tetracyclines (minocycline and tetracycline).

### 3.3. Whole genome sequencing

We set out to study the resistome using a collection of 23 type strains for the most abundant species that were uncovered by our extensive bacteriome analysis. For fifteen of these strains, no complete genome was available, and we thus set out to *de novo* assemble their complete genomes as a reference for the community and to avoid missing antibiotics resistance-relevant genes in fragmented Illumina assemblies (Varadarajan et al., 2020). A combination of long reads from third generation long read sequencing platforms (PacBio or ONT) and short read Illumina sequences (for polishing and to identify potential small plasmids) was used and *de novo* assembled (Supplementary Methods). All 350 isolates were sequenced with Illumina HiSeq (Supplementary Methods). A first reference-based assembly was done using the 23 complete genomes of the respective type strain of each species as a reference. The median, minimum, and maximum coverage and median total sequence lengths are listed in Supplementary Table S6. Importantly, for the type strains, we also tried to assemble plasmid sequences (Supplementary Methods; Supplementary Table S2).

### 3.4. Prevalence of antibiotic resistance genes

**Figure 5:**
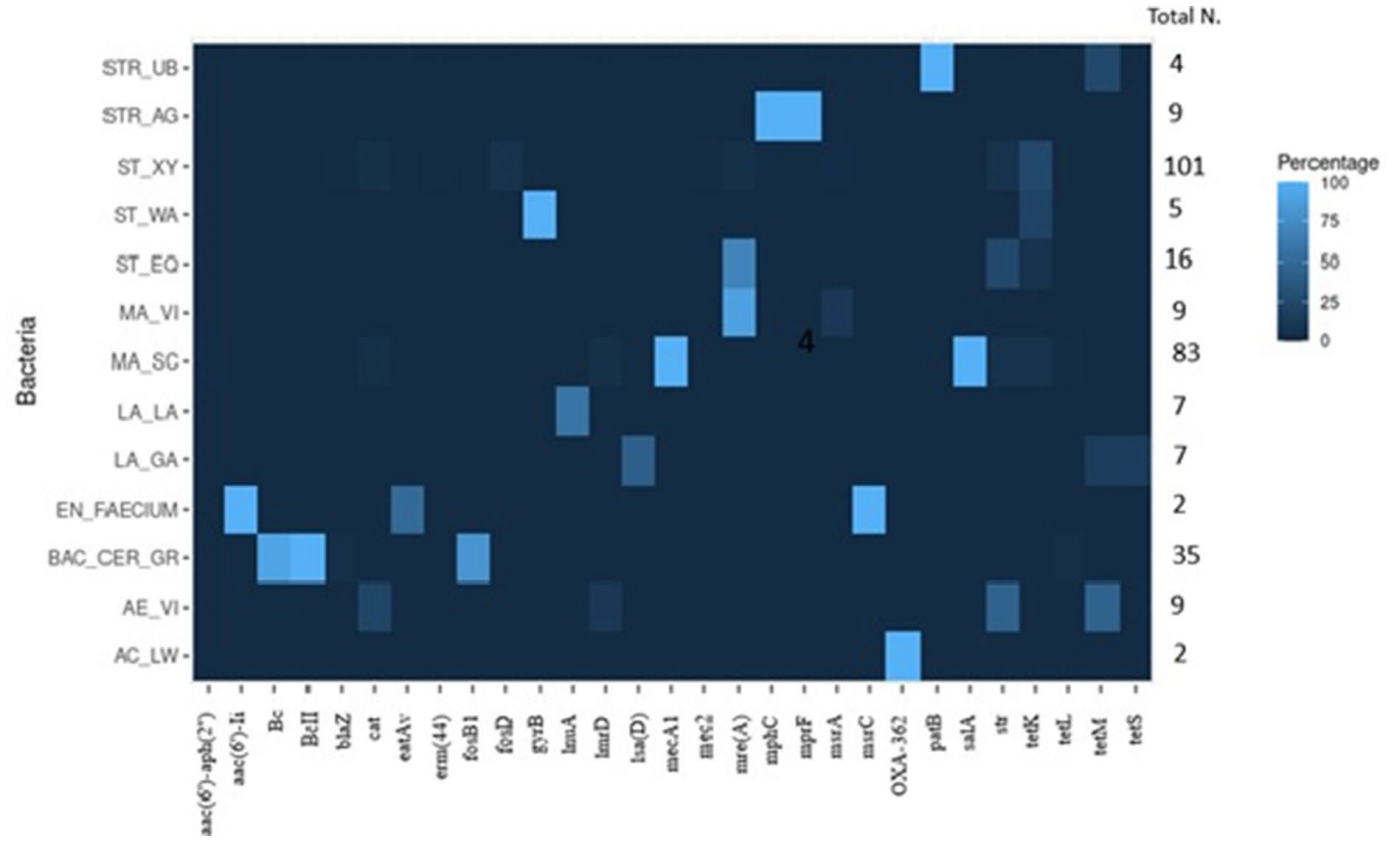
Graphical heatmap showing the results displayed in the Table 5. The different color scale represents the positivity of isolates resistant to the different ARGs.

To assess the presence and prevalence of ARGs, three different bioinformatic methods were applied. Combining the results of all methods, 66% of the 350 isolates carried at least one ARG. In total, 96 different ARGs were detected; based on the literature, 53 genes were classified as specific for resistance against one antibiotic molecule, 43 were implied in causing resistances to more than one molecule. The high number of identified ARGs largely results from the big number of genes detected by the CARD database for *S. aureus* and *E. coli* species. The complete results for the ARGs, including the functionality of the main ARGs detected in the isolates (Supplementary Tables S8, S9), can be found in the Supplementary material. The main ARGs detected in the 350 isolates are summarized in Table 5 and Figure 5.

**Table 5:** Main antibiotics resistance genes (ARGs) identified.

Bacteria	*aac(6′)-aph(2″)*	*aac(6′)-Ii*	*Bc or BcII*	*blaZ*	*cat*	*eatAv*	*erm(44)*	*fosB1*	*fosD*	*gyrB*	*lnuA*	*lmrD*	*lsa(D)*	*mecA1*	*mecC2*	*mre(A)*	*mphC*	*mprF*	*msrA*	*msrC*	*OXA-362*	*patB*	*salA*	*str*	*tetk*	*tetL*	*tetM*	*tetS*	Total N. Strains
ST_XY				1	4		1		6						1		4		1					6	**25**				101
MA_SC	1				3						3			**83**									**83**	5	5	2			83
ST_EQ																	**11**							4	1				16
MA_VI																	**8**		1										9
ST_WA										**5**															1				5
AC_LW																					2								2
AE_VI					2						1													4			4		9
BAC_CER_GR			35	1				**28**																		1			35
EN_FAECIUM		**2**				1														**2**									2
LA_GA													**3**														1	1	7
LA_LA												**4**																	7
STR_AG																**11**		**11**											11
STR_UB																						**4**					1		4

The corresponding phenotypic antibiotic resistance correlated with the genes are listed in Supplementary Table S7. The isolates *Staphylococcus succinus*, *Staphylococcus haemolyticus*, *Staphylococcus chromogenes*, *Arthrobacter gandavensis*, and *Enterococcus saccharolyticus* were negative for the detection of ARGs. The isolates with more than three ARGs [*Staphylococcus aureus* (ST_AU), *Enterococcus faecalis* (EN_FAECALIS), and *Escherichia coli* (ES-COLI)] are described in detail in the Supplementary Table S7. The bold values represent the majority of the strains carried the antimicrobial resistance genes.

#### 3.4.1. *Staphylococcus* spp.

For *S. xylosus*, 64 isolates (63%) were negative for ARGs, while in the 37 remaining isolates (37%) at least one ARG was detected. The *tetK* gene was the most prevalent gene observed in 25 isolates (25%). Other less prevalent genes were *fosD* (6 isolates), *str* (6), *cat* (4), *mphC* (4), *erm* (44) (1), and *msrA* (1). One isolate carried the ARGs *blaZ* and *mecC2*. The isolates of *S. chromogenes*, *Staphylococcus haemolyticus*, and *S. succinus* did not carry any known ARG. For *S. equorum*, most of the isolates (13%) harbored the *mphC* gene, in three isolates (19%) the *str* gene was detected. In one isolate *mphC*, *str* and *tetK* were observed simultaneously. *S. warneri* isolates were always positive for *gyrB*.

#### 3.4.2. *Mammaliicoccus* spp.

In all 83 *M. sciuri* isolates subjected to WGS, the *mecA1* and *salA* genes were detected. Additionally, two isolates (~2%) carried *cat*, *lnu (A)*, *str*, *tetK* and *tetL*. Only one isolate carried 6 ARGs: *mecA*, *salA*, *aac (6′)-aph (2″), lnuA, str* and *tetL*. Regarding *M. vitulinus,* seven out of nine isolates (77.8%) were *mphC* positive. One isolate additionally carried the *msrA.* Additional bioinformatic promoter analyses for the *mecA1* gene did not reveal any mutation in the promotor region in any of the 20 analyzed isolates when compared to the promoter region of the *M. sciuri* type strain (Wu et al., 2001; Frey et al., 2013).

#### 3.4.3. *Bacillus cereus* group

A high prevalence of β-lactams antibiotic resistance genes was detected in the *Bacillus cereus* group. The *Bc* gene was found in 24 isolates (68.6%) and the *BcII* gene that encodes a zinc metallo β-lactamase was found in all the isolates. In addition, most of the isolates were positive for *fosB1* (77.1%). In one case, the tetracycline resistance gene, *tetL*, was also detected.

#### 3.4.4. Farm-associated bacteria

Further analyses of the ARGs were done with the other 11 bacterial species, which were less prevalent in the milk samples. For *Arthrobacter gandavensis* and *Enterococcus saccharolyticus* bacteria, no ARGs were detected. The investigation of the *E. coli* isolates showed different results depending on the bioinformatics tools used. The analysis done with the ResFinder software revealed the presence of *mdfA* in all of the isolates. In one isolate, this gene was present together with *fosA7*. Notably, the analysis performed with the CARD database showed a different picture; more than 30 different genes were found in all 13 *E. coli* isolates and they seemed to be an intrinsic part of the *E. coli* genome but, based on our phenotypic profiling, not always expressed in the bacterial isolates.

For the other bacteria that belong to the bacteriome but that were less prevalent, a more detailed analysis for look regarding the presence of ARGs is explained in the Supplementary Table S8.

#### 3.4.5. Mastitis pathogens

For all streptococci, at least one ARG was present. All the *S. agalactiae* isolates carried *mre(A)* and *mprF*. All isolates of *S. uberis* carried the *patB* gene and in one isolate *tetM* was additionally observed. Some *S. aureus* isolates carried at least four resistance genes *mepR*, *mgrA*, *arlR*, and *glpT* (three isolates). All nine *S. aureus* isolates were positive for *arlR*, *mgrA* and *mepR*. In the remaining six isolates, more than four ARGs were detected. Only two isolates were positive for *blaZ*, while three different ARGs were detected specifically for fosfomycin resistance (*fosB, glpT*, and *murA*).

**Figure 6:**
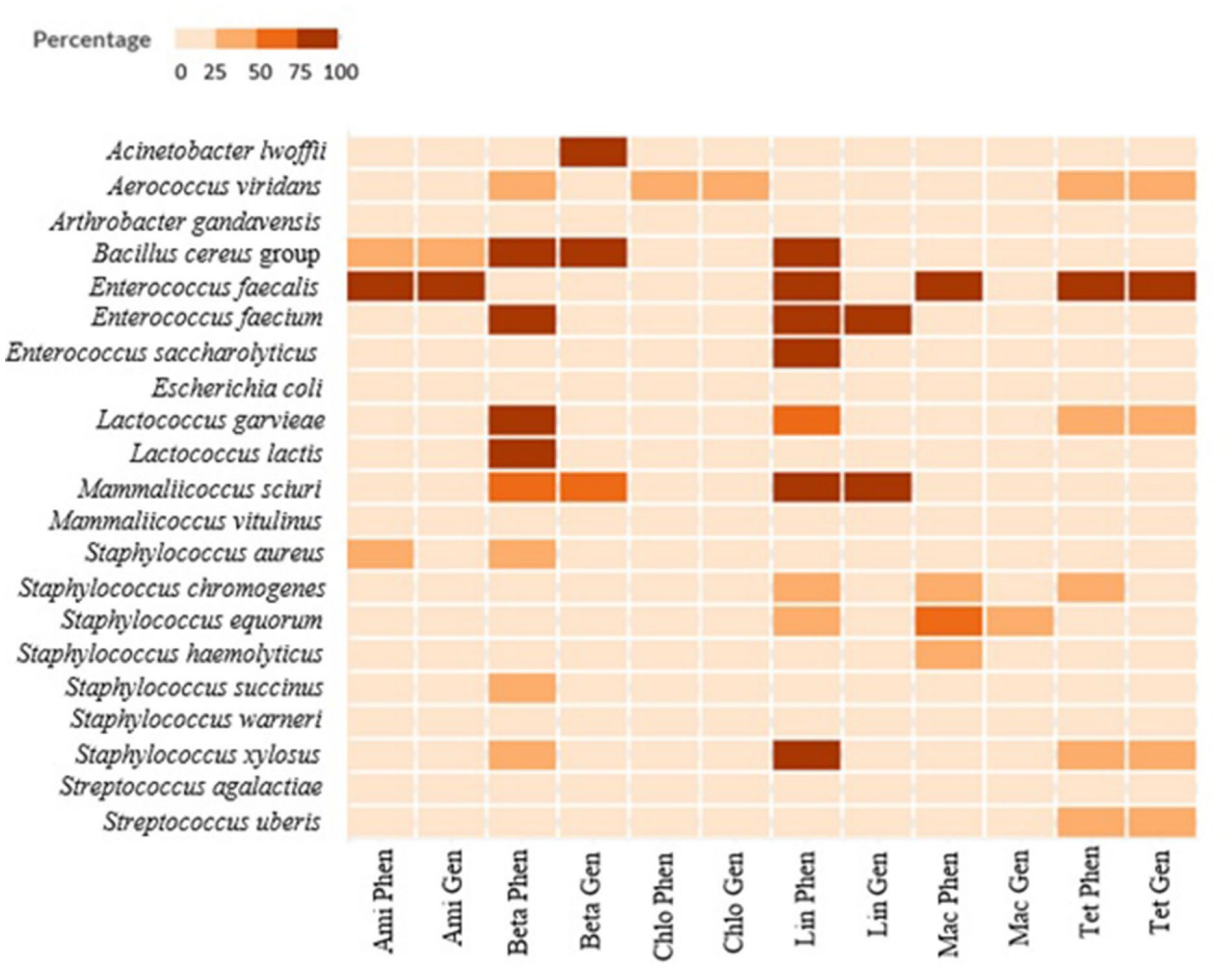
Correlation between phenotypic AMR resistance (in percent) and underlying genomic information for six classes of antibiotics (aminoglycosides, β-lactams, chloramphenicol, lincosamides, macrolides, and tetracyclines). The table displays the percentage of resistant isolates (phenotypic analysis) compared with the number of isolates where the ARGs correlated to the resistance to the antibiotics were identified. Ami Phen, Aminoglycoside phenotypic percentage; Ami Gen, Aminoglycoside genomic percentage; BetaPhen, β-lactams phenotypic percentage; Beta Gen, β-lactams genomic percentage; Chlo Phen, Chloramphenicol phenotypic percentage; Chlo Gen, Chloramphenicol genomic percentage; Lin Phen, lincosamides phenotypic percentage; Lin Gen, lincosamides genomic percentage; Mac Phen, Macrolides phenotypic percentage; Mac Gen, Macrolides genomic percentage; Tet Phen, tetracycline phenotypic percentage; Tet Gen, tetracycline genomic percentage.

A graphical heatmap shows the discrepancy between phenotypic resistant and the presence of ARGs in the same isolates (Figure 6). The results showed for several classes of antibiotics a low correlation between phenotypic and genomic results (aminoglycosides and β-lactams). Differently, in all the phenotypically resistant isolates the presence of the tetracycline ARGs was detected.

### 3.5. ARGs and plasmids

After *de novo* assembly, 104 (30%) out of the 350 isolates examined were positive for at least one plasmid-based replicon (*rep*) gene, in 40 cases (39%) the gene was located on a contig harboring at least one ARG (Table 6). In *S. xylosus*, *rep7* was mainly found in contigs carrying ARGs for tetracycline (*tetK*), chloramphenicol (*cat*), and streptomycin (*str*). Association of *rep7* with ARGs was also detected in *M. sciuri* (*str*, *cat*, *tetK*, and *lnuA*), in *S. equorum* (*str*, *str* plus *tetK*), and in *S. warneri* (*tetK*). Additionally, *rep7* was detected in three of nine *Aerococcus viridans* isolates that carried as well the *str* and *cat* gene. In *M. sciuri*, the gene *lnuA* was associated with 3 different *rep* types: *rep7*, r*ep13*, and *rep21*. *Rep1*3 was also observed in one contig (each) of *M. sciuri* and *S. aureus* carrying the *str* and *ermT* genes, respectively. The search for the *rep* genes was performed using PlasmidFinder. For comparison, identical analyses were also executed using PLSBD. In this case, 37 (35.6%) out of the 104 *rep* genes detected by PlasmidFinder were found.

**Table 6:** Results from the plasmid identification with PlasmidFinder (Center for Genomic Epidemiology, 2019).

Bacterial isolates	N. isolates positive (% on the total)	N. replicons	N. Isolates	Replicon proteins (number of isolates identified)
*Staphylococcus xylosus*	30 (30%)	1	27	***rep7a*** (15), *rep19c* (9), *rep 21* (3)
		2	3	***rep7a***, *rep19c* (2),***rep7a***, *rep21* (1)
*Mammaliicoccus sciuri*	17 (20%)	1	14	***rep7a*** (8), *rep13* (4), *rep21* (2)
		2	3	***rep7a****, rep13* (3)
*Staphylococcus succinus*	10 (45%)	1	9	*rep16* (5), *rep19c* (2), *rep20* (1), *rep21* (1)
		2	1	*rep16, rep21* (1)
*Staphylococcus equorum*	7 (43%)	1	7	***rep7a*** (4), *rep16* (1), *rep19* (2)
*Staphylococcus aureus*	6 (67%)	1	5	***rep7a*** (4), *rep13* (1)
		2	1	*rep16, rep19c* (1)
*Mammaliicoccus vitulinus*	1 (11%)	1	1	*rep19b* (1)
*Staphylococcus warneri*	1 (20%)	1	1	***rep7a*** (1)
*Aerococcus viridans*	3 (33%)	1	3	***rep7a*** (3)
*Bacillus cereus* group	1 (3%)	1	1	*rep22* (1)
*Enterococcus faecalis*	1 (100%)	4	1	*rep2, rep8b, repUS11, repUS41* (1)
*Enterococcus faecium*	2 (100%)	1	1	*rep1* (1)
		2	1	*rep1, repUS15* (1)
*Enterococcus saccharolyticus*	5 (83%)	1	4	*rep1* (2), *repUS21* (2)
		2	1	*rep1, repUS21* (1)
*Escherichia coli*	10 (77%)	1	2	*IncFCI* (2)
		2 3	4 4	*IncFIA, IncFIB* (2), *IncFIA, IncFII* (1), *IncX1, IncFIC* (1) *IncFIA, IncFIB, IncFII* (3), *IncFIB, IncFIC, Inc1-I* (1)
*Lactococcus garvieae*	3 (43%)	1	3	*rep32* (1), *rep33* (2)
*Lactococcus lactis*	5 (71%)	1	5	*repUS33* (5)
*Streptococcus uberis*	1 (25%)	1	1	*repUS43* (1)

For all NASM isolates phenotypically resistant to tetracycline including *S. xylosus* (*n* = 21), *M. sciuri* (*n* = 5), *S. equorum* (*n* = 1), and *S. warneri* (*n* = 1), a small plasmid could be identified always carrying the same *tetK* gene (100% similarity among strains) and the same *rep7a* replication initiation factor gene (Supplementary Table S10). Two of four plasmids circularized with plasmid Spades and Unicycler were, with 4,440 bp and 4,439 bp, very similar in size and were both composed by the three genes *rep7a*, *tetK*, and a plasmid recombination gene (5′-3′). The similarity was 99.7%. The other two circularized plasmids were larger in size amounting to 4,666 bp and 4,804 bp. They showed the same gene structure as the smaller plasmids, but the non-coding part was larger. The remaining 24 plasmids matched best with the 4,440 bp circularized plasmid (= reference) and showed a size between 4,435 bp and 4,448 bp; 19 of them exhibited a size of 4,440 bp with a similarity between 98.7 and 100.0% (toward the reference). Interestingly, 13 of those plasmids showed an identical similarity of 99.7% when compared to the reference. They were mostly observed in *S. xylosus* (*n* = 11), but also in *M. sciuri* (*n* = 1), and *S. warneri* (*n* = 1) and were repeatedly found in isolates within different farms.

## 4. Discussion

The composition of the intramammary bacteriome of healthy, untreated cows is extremely relevant in order to assess the prevalence of commensals and potential pathogens and to determine what kind of antibiotics resistance traits are encoded by these species. Enabled by large advances in next generation sequencing, we here describe the most extensive bovine intra-mammary bacteriome study to date, which we expect to have far-reaching implications for veterinary bacteriology practice and for diagnostics (Figure 1). These aspects are becoming increasingly important in the context of the One Health concept (McEwen and Collignon, 2018), where a large reduction of the use of antibiotics is envisaged, including their wide-spread use in animal husbandry and livestock production. Moreover, knowledge about the routes of transmission of mobile antibiotics resistance elements from animals to humans is also critical. To extend on the very detailed bacteriome, we thus also analyzed 350 isolates from the most abundant species by WGS [informed by a collection of 23 type strains, 15 of which we here assembled *de novo* relying on our expertise (Schmid et al., 2018)] and the knowledge that, compared to complete genomes, Illumina assemblies can miss genes relevant for antibiotics resistance (Varadarajan et al., 2020) and phenotypic profiling. This was done in order to (i) determine their resistome and (ii) to attempt to link phenotypic profiling data with antibiotics resistance genes, an area that requires further developments including more comprehensive and better curated databases of ARGs.

The current study highlights the presence of mainly NASM bacteria in mammary glands from healthy cows of nine Swiss herds. Overall, the bacterial isolates were found to be highly resistant to clindamycin and oxacillin. Genomic analyses revealed some consistent patterns regarding the presence of antibiotic resistance genes, for example the presence of *mecA1* and *salA* in all *M. sciuri* isolates. The tetracycline resistance was related to *tetK,* encoded on a small plasmid, which implicated a possible horizontal gene transfer between different NASM. For various phenotypic AB resistances observed, however, no ARGs were detected. As a consequence, further analyses should be performed to identify new ARGs or *in vitro* studies regarding the antibiotic resistance of veterinary bacterial isolates to actualize the current breakpoint of the MIC broth microdilution.

### 4.1. Bacteriome

In recent years, a few studies have investigated the bovine intramammary bacteriome using culture-dependent and culture-independent approaches (metagenomics) (Oikonomou et al., 2014; Cremonesi et al., 2018; Metzger et al., 2018; Sartori et al., 2018; Al-Harbi et al., 2021). Based on their findings, the bovine mammary gland is considered to contain a spectrum of different bacterial species. The results of the present study, using a culture-dependent approach, support these recent findings, but they show a much broader and more complex diversity of bacteria than expected. Bacteria from the NASM (61.1%) and the *Bacillus cereus* group (9%) were most frequently identified. Bacteria commonly present in the farm environment were less abundant [*Acinetobacter* spp. (1.8%), *Aerococcus viridians* (1.6%), *Arthrobacter* spp. (1.9%), *Enterococcus* spp. (3.2%), *E. coli* (2.2%), and *Lactococcus* spp. (1.9%)]. Further, a low percentage of known mastitis pathogens were detected in healthy cows [*S. aureus* (2.4%), *S. agalactiae* (0.8%), and *S. uberis* (1.3%)]. Indeed, in the present study 15.5% of the analyzed quarters showed a subclinical mastitis as defined by IDF (SCC ≥ 200,000 cell/mL) and these pathogens definitely contributed to the prevalence although it was also observed in quarters with monocultures of *S. xylosus* (19%) and *M. sciuri* (8%) (International Dairy Federation, 2022). Rarely, increased SCC were also observed for other NASM and bacteria and for co-cultures (data not shown). For *S. xylosus*, its prevalence is substantial, whereas for *M. sciuri* it is lower.

The present study further demonstrates that the intramammary presentence of *S. xylosus* and *M. sciuri* is highly farm dependent but is independent on the cow’s stage and number of lactations at the cow level. At the quarter level, however, a significant association was established for *S. xylosus* and both variables indicating that quarters of older cows and in progressed lactation are more susceptible to intramammary *S. xylosus*. For *M. sciuri*, a significant association between stage of lactation and intramammary presence was observed at the quarter level demonstrating that intramammary *M. sciuri* is more common during later lactation.

#### 4.1.1. Non-*aureus* staphylococci and mammaliicocci

*Staphylococcaceae* represented by *S. xylosus* (25%) and *M. sciuri* (20.6%) were the most frequently detected bacteria, followed by *S. succinus* (5.7%). Interestingly, *S. xylosus* and *M. sciuri* were frequently (40%) co-isolated from the same quarter. These results confirm previous studies where *S. xylosus* and *M. sciuri* were also the most frequently isolated bacteria (Malinowski et al., 2011; Frey et al., 2013; Xu et al., 2015; De Visscher et al., 2016; Condas et al., 2017; Sartori et al., 2018; Valckenier et al., 2020). In the present study, however, with 73.5% of milk quarter samples being positive for NASM, the detection rate was higher, particularly when compared to studies that have analyzed milk samples from healthy cows (Porcellato et al., 2020; Al-harbi et al., 2021). It has been shown that the prevalence and distribution of NASMs is influenced by regional and environmental factors (Vanderhaeghen et al., 2015; Rowbotham and Ruegg, 2016; Alanis et al., 2021). Indeed, we detected, a clear association between intramammary bacteria and the bedding: mammary glands of cows kept on straw contained a higher number of NASM; while in one farm, in which manure was used as bedding, *Enterobacteriaceae* and *Enterococcaceae* were predominant. However, this finding needs further investigations.

#### 4.1.2. *Bacillus cereus* group

An important aspect is the high prevalence of bacteria belonging to the *Bacillus cereus* group in milk samples of healthy cows (9%). This bacterial group, including twelve closely related species, is commonly found in environmental and food products accounting for between 11 to 47% of isolates, particularly in raw milk from cows and buffalo (Liu Y. et al., 2015; Owusu-Kwarteng et al., 2017; Baldwin, 2020; Radmehr et al., 2020; Zhao et al., 2020; Bartoszewicz and Czyżewska, 2021). Due to heat-resistant spores, these bacteria survive the pasteurization process and could cause spoilage of dairy products and even intoxication of human consumers (Gopal et al., 2015). All of these strains carried multiple ARGs.

#### 4.1.3. Farm associated bacteria

The prevalence of these bacteria was low and accounted for a range between 1.6 to 3% of all isolates. They included *Acinetobacter lwoffii, Arthrobacter gandavensis, E. coli, Enterococcus faecalis, Enterococcus faecium, Enterococccus saccharolyticus, Aerococcus viridans, Lactococcus garvieae* and *Lactococcus lactis*. *E. coli* and enterococci are typical fecal representatives [National Mastitis Council (NMC), 2017], whereas lactococci are commonly isolated from raw milk with the ability to persist in a farm environment and the cows (Werner et al., 2014). In addition, *Lactococcus garvieae* and *Lactococcus lactis* were previously identified as pathogens inducing chronic subclinical mastitis in cows, mostly during late lactation (Wyder et al., 2011). *Acinetobacter lwoffii* is mainly isolated from human skin and infections, from soil and plants, but also from other sources (Adewoyin and Okoh, 2018). In contrast, *Arthrobacter gandavensis* and *Aerococcus viridans* are largely found on bovine teat skin and in milk (Wyder et al., 2011; Verdier-Metz et al., 2012). These results demonstrate that farm-associated bacteria can be part of the intramammary bacteriome, but compared to NASM, they play a minor or even a negligible role.

#### 4.1.4. Mastitis pathogens

With an observed frequency of 2.8%, mastitis pathogens were rarely observed. Their presence was farm specific (especially for Farm 4) and included *S. aureus*, *S. agalactiae*, and *S. uberis*. For the streptococci, our findings were very similar to a previous Swiss study that had screened whole herds for *S. aureus* (Moret-Stalder et al., 2009). The low presence of pathogens in the present study is not astonishing as only healthy cows with healthy udders were included, whose milk was suitable for human consumption according to Swiss legislation. Nevertheless, they were detected, raising the question why they were observed. All observed bacteria are known to be frequently involved in subclinical bovine mastitis [National Mastitis Council (NMC), 2017], meaning that the udder and milk are grossly normal so that the farmer was unaware that an IMI was present. Alternatively, it may be possible that the isolates represent apathogenic subtypes, as has been previously shown for *S. aureus* (Fournier et al., 2008).

### 4.2. Clinical and diagnostic implication

For decades, veterinarians have been convinced that the bovine mammary gland is sterile, and all bacteria isolated from milk have been considered a result of an intramammary infection and mastitis. This has been increasingly questioned in recent times, when it turned out that particularly NASM could regularly be isolated from milk samples of the same, healthy quarter over time (Sartori et al., 2018). So far, NASM have been considered as minor pathogens causing subclinical forms of mastitis (Sears and McCarthy, 2003; Taponen and Pyörälä, 2009; Condas et al., 2017) and are the most common bacteria isolated from clinical milk samples sent in for diagnostic analysis by veterinarians [National Mastitis Council (NMC), 2017]. NASM infections are regularly treated with AB, at least in Switzerland, based on the assumption that the bovine mammary gland is free from bacteria. In contrast to almost all other studies, and certainly in contrast to the clinical work in the field where milk samples are only taken and bacteriologically examined after an udder health problem has been detected, the present study, however, focuses on healthy quarters. Importantly, the same standard diagnostic culturing methods were used as they are routinely applied. And suddenly the very same NASM were found at the species level, a fact that definitely questions the role of NASM in the context of bovine mastitis. Are they really minor pathogens or do they have a more protective function? Even the finding that NASM are found together with increased amounts of inflammatory cells in the milk does not necessarily mean that they are the cause of the observed inflammation. It is well known that inflammation of the mammary gland can also result from inappropriate milking procedures leading to mechanical tissue irritation (Giesecke et al., 1986), the NASM could merely be a by-product as, at the time of sampling, they were in the quarter anyway. As a consequence, and clearly based on the present study, it is no longer possible to interpret the finding of NASM isolated from a diseased quarter in clinical terms. This is particularly true for *S. xylosus* and *M. sciuri* which first of all had been both isolated from healthy quarters. Under field conditions, a positive culture for these bacteria should no longer be interpreted in the way that a subclinical mastitis is present in the corresponding quarter. This is only possible if the milk of the quarter shows SCC ≥ 200,000 cells/mL or a positive California mastitis test.

Indeed, the clinical significance of NASM present in the mammary gland remains to be further elucidated. Potentially, the most abundant NASM such as *M. sciuri* and *S. xylosus* need to be further subtyped to tease out some relevant differences.

### 4.3. Resistome

In Switzerland, between 2018 and 2019 approximately 70% of all antimicrobials used for IMI were products applied for the treatment of mastitis during lactation. Penicillin followed by aminoglycosides are the most predominantly used antibiotics according to the Swiss Antibiotic Resistance Report 2020 (Federal Office of Public Health and Federal Food Safety and Veterinary Office, 2020). Over the last years, an increase of antimicrobial resistant bacteria has been reported which is at least in part due to the misuse of antibiotics in agriculture (Manyi-Loh et al., 2018; Mann et al., 2021). Concomitantly, ARGs were detected in different environments including milk samples (Pol and Ruegg, 2007; Saini et al., 2013).

#### 4.3.1. *Staphylococcus* spp.

Although their pathogenicity and epidemiology are still under debate (Nyman et al., 2018; De Buck et al., 2021), IMI caused by NASM are regularly treated with antibiotics in Switzerland (BLV, 2019). This requires a good understanding of AB resistance patterns and the mechanisms of action to offer an optimal therapy. In this study, they exhibited the highest resistance to clindamycin and oxacillin, which has increased compared to a previous Swiss study (Frey et al., 2013). The reason of the clindamycin resistance increase could be the recent decrease and change in the resistance breakpoint (from >0.5 to >0.25 mg/mL) in the EUCAST guidelines [The European Committee on Antimicrobial Susceptibility Testing (EUCAST), 2022]. The percentage of penicillin resistant isolates was higher (23.3%) than the percentage for the *Staphylococcus* spp. isolates in the Frey work (8%). Both studies from Switzerland recognized a high number of isolates resistant to oxacillin, 20 and 47%, respectively. A tetracycline resistance rate between 12 to 38% was detected in *S. xylosus* bacteria in a previous Swiss study of different food products such as fermented sausages, cheeses, and meat starter cultures (Leroy et al., 2019). Our study, with a percentage of 25% *S. xylosus* isolates, confirm a high resistance prevalence for this antibiotic.

#### 4.3.2. *Mammaliicoccus* spp.

Previous studies involving *Mammaliicoccus* spp., showed a high variability of tetracycline resistant isolates. In Switzerland, two previous studies analyzed the presence of *M. sciuri* in pigs, cattle, poultry, and different food matrixes as bulk tank milk, minced meat, and abattoir employees demonstrated the presence of resistant *M. sciuri* (Huber et al., 2011; Nemeghaire et al., 2014). The low resistance to aminoglycosides found in our study, agrees with a study by Hauschild et al. (2007) where the resistance of 204 *M. sciuri* isolates was evaluated. Recently, Lienen et al. (2022), revealed a high resistance prevalence of 26 *M. sciuri* isolates against five to twelve antimicrobial substances including methicillin. Additionally, a high prevalence of penicillin resistance was detected (90%), much higher than in our study (52%). The high number of resistant isolates can be attributed to a selection of the *Mammaliicoccus* isolates belonging to herds where MRSA isolates were detected. Interestingly, 15% of *S. xylosus* and 21% of *M. sciuri* were MDR. The prevalence of multiple resistant isolates was considerable and can become problematic, if the ARGs would be transferred to a pathogenic bacterium such as *S. aureus.*

#### 4.3.3. *Bacillus cereus* group

The *Bacillus cereus* group showed a high prevalence of resistance to β-lactams, in accordance with a recent paper (Bartoszewicz and Czyżewska, 2021) that had isolated *Bacillus* isolates from raw milk and which showed more often resistance than isolates from natural environments; the authors hypothesized that the higher resistance could be due to residual amounts of antibiotics in the milk. To our knowledge, and as described before, a high antibiotic resistance could be associated with intrinsic phenotypic resistance (Owusu-Kwarteng et al., 2017; Mills et al., 2022).

#### 4.3.4. Farm-associated bacteria

The two *Acinetobacter* isolates analyzed in our study were sensitive to all antibiotics and did not show any known ARGs. In contrast, Gurung et al. (2013) found that *Acinetobacter* were highly resistant to different antibiotics tested.

The nine *Aerococcus viridans* isolates were mainly resistant to β-lactams (5) and tetracycline (4). These results partially agree with those of Sun et al. (2017) showing only a partial resistance for tetracycline and no resistance for the β-lactams. A high resistance prevalence to trimethoprim/sulfamethoxazole was found in previous works (Martín et al., 2007; Liu G. et al., 2015; Sun et al., 2017) while in this study, only one isolate was resistant to this antibiotic. Notably, the differences between the studies could be due to the different methods used; the earlier studies mainly used disk diffusion while our study used the microdilution broth (MIC).

The Enterococci of the present study (*Enterococcus faecalis, Enterococcus faecium, Enterococcus saccharolyticus*) were commonly resistant to lincomycin. This is in line with the results described by Różańska et al. (2019). All *Enterococcus saccharolyticus* and all *Enterococcus faecalis* were resistant to synercid. No isolates were resistant to vancomycin, which represents an example of an actual increasing clinical problem in humans with infections caused by *Enterococcus* spp. Our enterococci isolates showed less antibiotic resistant isolates compared to those isolated from humans (Rogers et al., 2021). This is most likely due to the fact that the majority of enterococci found in our study were *Enterococcus saccharolyticus,* which were susceptible to all AB tested except to lincosamides and synercid.

A general low resistance prevalence to antibiotics was found in *E. coli* isolates in this study (three isolates out of 13). Colistin resistance, a last resort antibiotic, was found in two isolates. This is in contrast to the Swiss antibiotic report (Federal Office of Public Health and Federal Food Safety and Veterinary Office, 2020) where all *E. coli* isolates causing bovine mastitis were susceptible to this AB.

Most *Lactococcus* spp. isolates, were multidrug-resistant. This result was in contrast to the Swiss study by Walther et al. (2008), who demonstrated that *Lactococcus lactis* isolates were sensitive to all 17 antibiotics tested. In that study, the main resistances were observed to tetracycline, clindamycin, and erythromycin, respectively (14.6, 7.3, and 7.3%). In contrast, our work showed a high resistance to rifampin (100%), penicillin, and cephalosporins (71%). Another *Lactococcus* species, *Lactococcus garvieae*, exhibited resistance to clindamycin (four out of seven isolates). Additionally, resistance to rifampin and β-lactam AB was prominent, disagreeing with previous Swiss, and international studies (Walther et al., 2008; Devirgiliis et al., 2013). However, the higher β-lactams resistance observed in the current study could well be associated with the large use of this class of antibiotics at the intramammary level.

#### 4.3.5. Mastitis pathogens

The resistance profile of bovine streptococci has been shown to be strongly influenced by the geographical origin of the sample and the species (Saed and Ibrahim, 2020; Kabelitz et al., 2021). In our study, all *S. agalactiae* isolates were sensitive to all the antibiotics used in the MIC assay. This supports the results of a recent study of bovine mastitis isolates in which the resistance to antibiotics in *S. agalactiae* isolated from different European countries were studied (El Garch et al., 2020). The tetracycline resistance rate of *S. uberis* agreed with the results obtained by El Garch et al. (2020) and with Swiss data (Federal Office of Public Health and Federal Food Safety and Veterinary Office, 2020). Interestingly, although penicillin is the most frequently used antibiotic against *Streptococcus* causing mastitis, no resistant isolates were found. This observation agrees with the work of Käppeli et al. (2019). The percentage of penicillin resistance of *S. aureus* isolates is comparable to results reported in two other European studies (El Garch et al., 2020; Ivanovic et al., 2023).

### 4.4. *In silico* analysis of ARGs and association with phenotypic results

Advances in DNA sequencing technologies and the availability of various bioinformatics tools and curated databases of ARGs have revolutionized diagnostic microbiology and microbial surveillance (Hendriksen et al., 2019). In our study, we used bioinformatics tools to try to find the ARGs causing the observed resistance. In particular, the genomes were evaluated for ARGs using two of the most popular online tools, Comprehensive Antibiotic Resistance Database (CARD) and ResFinder (Center for Genomic Epidemiology, 2019); they are very effective and have sustainable curation strategies (Lal Gupta et al., 2020). Additionally, an in-house, BLAST based program (GBlast) together with a manually curated database for *Staphylococcus* spp. containing 105 ARGs was used to specifically search for fragmented genes.

The present study demonstrates that there still is a big gap between the phenotypic and genotypic findings. In fact, for many bacteria and many ABs tested, no ARGs were found that could explain the observed phenotype. Obviously, the genetic basis of the mechanisms leading to phenotypic AMR in the bacteria present in the surrounding of cows is less well understood compared with the main pathogens causing mastitis. Nevertheless, *S. xylosus* plays an important role as a fermentative agent in food industry and *Bacillus cereus* has been known for its role in food poisoning for a long time. Particularly striking is the poor association between phenotype and genotype for NASM although recent genomics methods and databases were used. From an evolutionary point of view, NASM are close to *S. aureus*, which has been intensively investigated for AMR during the last decades (Madhaiyan et al., 2020). Based on these considerations, we expected to find the NASM chromosomal ARGs orthologous to those of *S. aureus* that contribute to intrinsic AMR. Furthermore, we expected to find the same plasmid based ARGs that were previously observed in *S. aureus,* as a possible plasmid transfer between the two species was observed (Fišarová et al., 2019). Except for *tetK* (see below), few of these assumptions turned out to be correct. The reasons for this discrepancy remain largely unclear. For orthologous genes, the similarity between those of NASM and *S. aureus* used as the target genes is probably too low so that the NASM genes cannot be detected by the software tools applied. Considering the plasmid transfer, it is rare between *S. aureus* and NASM in the environment of cows although *S. aureus* is a major mastitis pathogen and is commonly observed on dairy farms (Leuenberger et al., 2019). From a technical point of view, the discrepancy between phenotypic and genotypic findings is hardly the result of inappropriate genomic methods, since the same were successfully applied in our recent publication to predict phenotypic penicillin resistance in *S. aureus* by WGS (Ivanovic et al., 2023).

Importantly, the present work demonstrates that only using genomic approaches might not be adequate to infer phenotypic AMR. Additional genomic and wet laboratory investigations are necessary, and a more comprehensive overview of the mechanisms of actions of different antibiotics.

#### 4.4.1. *Staphylococcus* spp.

Except for tetracycline, no or little genomic information was found explaining the observed resistances in *Staphylococcus.* This is particularly true for β-lactam AB (penicillins and cephalosporins), lincosamides, and in part for macrolides (limited explanation for *S. equorum*). These results demonstrate that NASM may have developed their own mechanisms of resistance. Maybe some of these mechanisms rely on yet undiscovered genes orthologous to known ARGs, but it is also possible that they are based on completely distinct and so far unknown mechanisms and genes. Tetracycline resistance could be fully explained in all resistant isolates by the presence of the *tetK* gene. It was always present on a small plasmid (approx. 4,440 bp after closing) and displayed a 99% similarity to the plasmid pSX10B1, previously found in *S. xylosus* isolated from fermented sausage and linked to a possible, albeit low, transfer of this resistance to other *S. xylosus* strains (Leroy et al., 2019).

The missing association between phenotypic and genotypic results for β-lactam ABs has largely to do with the fact that no known ARGs were found explaining penicillin and oxacillin resistance in *S. xylosus* and *S. succinus*. In fact, no *blaZ* or *mecA, mecA1* or *mecC* genes were observed except in one case. The lack of these genes in *S. xylosus* agrees with the PCR findings reported by Frey et al. (2013), who were also unable to detect them. In the case of *S. equorum*, 75% of the isolates were positive for the *mphC* gene, explaining their observed resistance to macrolides. Although this association is substantial, still 30% of the isolates rely on genes other than *mphC.* Interestingly, this gene was located on the chromosome and was not associated with a mobile element suggesting that it is an intrinsic part of the *S. equorum* chromosome.

#### 4.4.2. *Mammaliicoccus* spp.

The *mecA1* and s*alA* genes were observed in all isolates analyzed. Both were located on the chromosome and were not associated with a mobile element. This means both genes are part of the core genome of *M. sciuri* and contribute to the intrinsic AMR of this bacterium. A high association was observed between clindamycin resistance (lincosamides) and the presence of *salA,* as all resistant isolates (93%) also carried the gene. In contrast, as previously found by Cai et al. (2021), although 100% of the isolates carried the *mecA1* gene, only 55% of the isolates showed resistance to oxacillin. For *M. sciuri* it is known that high-level *mecA1* expression is required to observe oxacillin resistance in these bacteria. Overexpression is associated with a *mecA1* promoter mutation at position-10 (Wu et al., 2001; Frey et al., 2013). In the present study, however, none of the 20 isolates analyzed showed the indicated mutation independent whether they were oxacillin resistant or not. Furthermore, all 20 genes could be translated *in silico* into a full-length protein suggesting that the protein function was not harmed by a mutation. These findings suggest that a different mechanism than the previously observed overexpression of *mecA1* accounts for the observed oxacillin resistance.

Even more controversial was that all oxacillin resistant isolates were penicillin susceptible although the low-affinity penicillin-binding proteins (PBP2A) encoded by the *mec* genes including *mecA1* cause resistance to all β-lactam AB (Schwendener and Perreten, 2022). All these findings suggest to re-consider the function of *mecA1* and its expression in future studies. The fact that resistance to β-lactam AB at the genomic level is still incompletely understood in *Staphylococcaceae* is further illustrated by our results for *M. vitulinus*: two isolates were oxacillin resistant, but no ARG was found. Considering tetracycline resistance and *M. sciuri*, all phenotypically positive isolates harbored either the *tetK* (*n* = 5) or the *tetL* genes (*n* = 2). The *tetK* gene was always found on the same small plasmid as observed in *S. xylosus*. For the *tetL* gene, a location on a new, larger plasmid or a correlation with a transposon could be assumed but this will require a further, detailed follow-up study.

#### 4.4.3. *Bacillus cereus* group

The majority of the *Bacillus cereus* group isolates contained *fosB1*; confirming previous reports that found this ARG in isolates from vegetables (Fiedler et al., 2019) and humans (Bianco et al., 2021). Just in one case we could find an association with the phenotypic results.

All *Bacillus cereus* isolates carried the gene *BcII*, previously shown to be involved in β-lactam resistance (Bartoszewicz and Czyżewska, 2021). Additionally, in agreement with the study of Fiedler et al. (2019), a lower prevalence of the *Bc* gene was detected (68%). In our study, when at least one of the two genes was present, the resistance to β-lactams could be always detected.

#### 4.4.4. Farm-associated bacteria

A recent review about the resistomes of *Acinetobacter* non-*baumannii* strains demonstrated that penicillin resistance mediating ARGs were described to be the most prominent resistance genes in these bacteria (Baraka et al., 2020). This study supports these results, with the presence of the gene *Oxa-362*.

A perfect agreement between some phenotypic results and the ARGs was found in *Aerococcus viridans*: all isolates were resistant to tetracycline and, concomitantly, the *tetM* gene was detected. The same was true for the chloramphenicol resistant isolates, which encoded the corresponding *catA8* gene. In contrast, no ARGs were found that could explain oxacillin resistance, and the presence of the *str* gene was not accompanied by streptomycin resistance.

With a total of 49 genes, a rather high number of ARGs were detected in *E. coli*. Thirty-one genes were observed in all isolates, a finding that confirmed the results of Tyson et al. (2015). In addition, 18 ARGs were found in 8 to 92% of the isolates. Despite the large number of ARGs, all isolates except three were susceptible to all ABs tested. This discrepancy could be easily explained by the fact that for clinical use, and implemented on the commercial resistance plate, only those AB classes for which inherent genes and AMR known mechanisms were contained. The lack of clinical relevance is probably also the reason why most ARGs found in CARD for *E. coli* were missing in the ResFinder database (Center of genomic Epidemiology), an observation that had previously also been made by Jeamsripong et al. (2021).

*Lactococcus garvieae* and *Lactococcus lactis* were mainly positive for the ARGs *lsa(D)* and *lmrD*, respectively. These genes confer resistance to lincosamides, streptogramins, and pleuromutilins (Shi et al., 2021). In the present study, however, both species were susceptible to these ABs. Further discrepancies between phenotype and genotype were also found for other ABs: although all *Lactococcus garvieae* and *Lactococcus lactis* isolates were resistant to rifampicins and the majority to cephalosporins, no ARGs were detected for both species.

#### 4.4.5. Mastitis pathogens

All *S. agalactiae* isolates carried the ARGs *mprF* and *mreA*. Both genes are commonly present in this species and are always found on the chromosome. *MprF* confers resistance to peptide-based AB while *mreA* to macrolides (Clancy et al., 1997; Ernst et al., 2009). However, in the present study, no phenotype antibiotic resistance results were observed. This fact could be correlated with an intrinsic presence of the genes on the chromosome.

In *S. uberis* isolates, a variant of the gene *patB* was found. In contrast, the *patA* gene, which is correlated with the mechanism of resistance to quinolone, was not identified (El Garch et al., 2010). As a consequence, the resistance to fluoroquinolones could not be detected.

In all *S. aureus* isolates*, arlR, arlS, lmrS, mepR, mgrA,* and *norA* were identified. All these ARGs are intrinsically located on the chromosome and are considered to contribute to basic AMR of *S. aureus* (CARD database). In the present study, however, none of the isolates showed the corresponding phenotype. This is emphasized in case of AMR to quinolones as each isolate harbored the ArlR/ArlS and the MgrA/NorA systems. The same was also true for the *lmrS* gene that was present in all isolates, but no AMR was observed for aminoglycosides, linezolid, macrolides, and phenicols. Additionally, four isolates were *fosB* positive, but sensitive to fosfomycin. In contrast, the observed AMR to penicillin/ampicillin and azithromycin/erythromycin could be explained by the presence of *blaZ* and *ermT* genes, respectively. In *S. aureus*, both genes are normally plasmid based (Kadlec and Schwarz, 2010; Ivanovic et al., 2023) leading to the question whether the current MIC methods are appropriate to assess AMR resulting from mechanisms whose ARGs are intrinsically located on the chromosome. Alternatively, the ARG may not be expressed in the media that we used. In general, transcriptomic or proteomic analyses of the expression levels of certain ARGs (and under different conditions or media) would add additional relevant information for the aim to be able to link genotype and phenotype data.

In summary, the association between phenotype and genotype is missing for various ABs analyzed in the present study. This was observed for β-lactam, lincosamide, and macrolide ABs where the gap can be highlighted. Reasons for this discrepancy may be that the orthologous ARGs of these less commonly studied bacteria were not detected by the current bioinformatics methods, that the corresponding proteins were not expressed, or that, as a consequence of convergent evolution, other AMR mechanisms than the known ones were involved in expression of the observed phenotype. Creating complete genome assemblies for the 350 isolates was beyond the financial possibilities of this study, and might contribute to the fact that some ARGs were missed as well.

### 4.5. Plasmids and their transfer of ARGs

Plasmids are an important source for the exchange of ARGs between different species and have been reported for *Staphylococcus* species (Malachowa and Deleo, 2010; Mlynarczyk-Bonikowska et al., 2022).

Based on the analyses of circular plasmids, we can contemplate a possible horizontal gene transfer mechanism, when *S. xylosus* and *M. sciuri* co-exist in the same farm. The bacteria apparently transfer small plasmids involved in the antibiotic resistance mechanisms as for tetracycline (*tetK*). The plasmid harboring the *tetK* gene was found in *S. xylosus* (approx. 4,440 bp) and displayed a 99% similarity to the plasmid pSX10B1, previously isolated in *S. xylosus* from fermented sausage (Leroy et al., 2019). In this study, the small plasmid mediated the mechanisms of mobilization, but was rarely transferred to others *S. xylosus* bacteria. The small plasmids are non-conjugative mobilizable, which means that they are not able to be transferred without some helper elements as conjugative plasmids or transposons (Francia et al., 2004; Ramsay et al., 2016). In our study, the completely identical small plasmid found in *S. xylosus* was isolated also from *M. sciuri* and one isolate of *S. warneri* and *S. equorum*. The presence of the same small plasmids circulating in different species could suggest an exchange of the plasmids between the four NASM species. Additional to the small plasmids carrying the tetracycline resistance, other plasmids, carrying *cat* (chloramphenicol resistance) and *str* (aminoglycosides resistance), for instance were detected. These results, although limited to only some of the bacterial species, highlight a possible transfer of ARGs mainly through small plasmids.

In summary, our study finds a high prevalence of bacteria in aseptically collected milk samples from healthy cows. The composition of the intramammary bacteriome displayed a farm-and bedding-dependency: the predominant isolated species were *S. xylosus* and *M. sciuri*, especially in herds that used a straw bedding system. The physiological significance of the NASM in the mammary glands, however, remains to be elucidated in further studies. In contrast, species belonging to the *Bacillus cereus* group or other mastitis pathogens were only rarely detected. It is essential to get more knowledge about the bacteriome of the mammary glands of healthy and diseased cows to understand and preserve the physiologically normal microbiota, hinder pathogens to gain a foothold and, in the long term, prevent the development and spread of resistances. Further studies addressing the phylogeny of the isolates from milk and herd environment need to be done to understand the origin of the isolates. NASM displayed individual species-specific ARG profiles. Not all phenotypic resistances were based on the presence of known ARGs. WGS represents an important tool for detecting ARGs but still needs to be associated with phenotypic analysis and with gene and/or protein expression analyses. Screening for new genes associated with AMR and an increase of the ARG databases will be essential, especially for the One Health concept.

## Data availability statement

The datasets presented in this study can be found in online repositories. The names of the repository/repositories and accession number(s) can be found below: All the reads of the different bacteria isolated were uploaded in NCBI under the Bioproject PRJNA859642, https://www.ncbi.nlm.nih.gov/bioproject/859642. The genome and the plasmids of the 15 de novo assembled type strains were uploaded in NCBI under the Bioproject PRJNA936091, https://www.ncbi.nlm.nih.gov/bioproject/936091. Our manually curated database of 105 Staphylococcus spp. ARGs is released as Supplementary material Data Sheet 2.

## Author contributions

HUG designed and wrote the initial project application. AR, LS, and MV performed the sampling of the herds. AR, II conducted experiments. JW and LE performed the SCC measurements. TS, MS, and CHA performed *de novo* genome assembly of the type strains (with long read data contributed by DF and JF), identified additional plasmids and closed them. AR and HUG analyzed the data. AR and HUG wrote the first draft of the manuscript with substantial input from CHA. LV, MD, and AS contributed to the conceptualization of the manuscript. All authors contributed to the article and approved the submitted version.

## Funding

This research work was founded by the Federal Food Safety and Veterinary Office (FSVO). Open access funding by Agroscope.

## Conflict of interest

The authors declare that the research was conducted in the absence of any commercial or financial relationships that could be construed as a potential conflict of interest.

## Publisher’s note

All claims expressed in this article are solely those of the authors and do not necessarily represent those of their affiliated organizations, or those of the publisher, the editors and the reviewers. Any product that may be evaluated in this article, or claim that may be made by its manufacturer, is not guaranteed or endorsed by the publisher.

## Footnotes

^1^ https://www.fedlex.admin.ch/eli/cc/2005/824/de

^2^ https://rast.nmpdr.org

^3^ https://cge.cbs.dtu.dk

^4^ https://card.mcmaster.ca

^5^ https://cge.food.dtu.dk/services/PlasmidFinder

^6^ https://ccb-microbe.cs.uni-saarland.de/plsdb

^7^ https://www.ncbi.nlm.nih.gov/bioproject/PRJNA936091

^8^ https://www.ncbi.nlm.nih.gov/bioproject/PRJNA859642/
