# Supplementary material for: Elucidation of the Bovine Intramammary Bacteriome and Resistome from healthy cows of Swiss dairy farms in the Canton Tessin
Source: Front Microbiol. 2023 Jul 31;14:1183018. doi: 10.3389/fmicb.2023.1183018 (PMC10425240; doi:10.3389/fmicb.2023.1183018)
Supplement: Supplementary file 3 [file Data_Sheet_2.docx]

Supplementary Material

**Elucidation of the Bovine Intramammary Bacteriome and Resistome from healthy cows of Swiss dairy farms in the Canton Tessin**

**A. Romanò^*^, I. Ivanovic, T. Segessemann, L. Vazquez Rojo, J. Widmer, L. Egger, M. Dreier, L. Sesso, M. Vaccani, M. Schuler, D. Frei, J. E. Frey, C.H. Ahrens, A. Steiner, H.U. Graber**

*** Correspondence:** Alicia Romanò
[alicia.romano@agroscope.admin.ch](mailto:alicia.romano@agroscope.admin.ch)

Table of contents

[Supplementary Methods 2](#_Toc129015670)

[1.1 Bacterial DNA extraction for sequencing and assembly of type strains 2](#_Toc129015671)

[1.2 Library preparation and sequencing 2](#_Toc129015672)

[1.2.1 PacBio long read sequencing 2](#_Toc129015673)

[1.2.2 ONT long read sequencing 3](#_Toc129015674)

[1.3 Genome Assembly 4](#_Toc129015675)

[1.3.1 Assembly strategies for the different type strains 4](#_Toc129015676)

[1.4 Short read polishing and assembly quality control 5](#_Toc129015677)

[1.5 Phenotypic Antimicrobial Susceptibility Testing 6](#_Toc129015678)

[1.6 References 7](#_Toc129015679)

[Supplementary Tables 9](#_Toc129015680)

[Supplementary Figure. 18](#_Toc129015681)

Supplementary data……………………………………………………………………………………………………………19

# Supplementary Methods

## Bacterial DNA extraction for sequencing and assembly of type strains

Eight complete genomes from a subset of the 23 type strains were downloaded from NCBI (see Table S1). The remaining 15 strains for which no complete genome was available at the time were ordered from the DSMZ (German Collection of Microorganisms and Cell cultures, Braunschweig, Germany) and sequenced with both Illumina (short reads) and PacBio/ONT (long reads) (Table S2). Different DNA extraction kits/protocols were used depending on the sequencing method (intact high molecular weight genomic DNA (gDNA) for long read sequencing) and the bacterial species.

For Illumina sequencing, the DNA of the type strains was extracted with the NucleoSpin® 8 Plasmid kit (Macherey-Nagel), and sequenced on a HiSeq sequencing platform (Illumina, San Diego, CA) at Eurofins Genomics GmbH (Ebersberg, Germany).

For Oxford Nanopore Technologies sequencing, gDNA from *Staphylococcus equorum*, *Staphylococcus succinus*, *Enterococcus faecium* and *Staphylococcus chromogenes* was extracted with the GenElute™ bacterial genomic DNA kit (Sigma-Aldrich).

For Pacific Biosciences SMRT sequencing, the DNA of the strains was extracted with two different protocols. GenElute™ bacterial genomic DNA kit (Sigma-Aldrich) was used to extract gDNA form the type strains *Staphylococcus haemolyticus*, *Mammaliicoccus vitulinus,* *Staphylococcus warneri*, *Arthrobacter gandavensis*, *Lactococcus lactis*, *Lactococcus garvieae*, *Acinetobacter lwoffii*, and *Enterococcus faecalis*. Alternatively, high molecular weight gDNA was recovered using rapid extraction with guanidium thiocyanate (Pitcher et al., 1989) for the type strains of *Bacillus mycoides*, *Enterococcus saccharolyticus*, and *Aerococcus viridans*. This extraction was performed by the Next Generation Sequencing Platform (University of Bern).

## Library preparation and sequencing

### PacBio long read sequencing

For PacBio long read sequencing, one ug of gDNA in 100 µL elution buffer was sheared using a Covaris g-TUBE (Covaris; 10145) and concentrated and cleaned using AMPure PB beads. The samples were then quantified and qualified to be in the range of 12-15 kb using a Qubit 4.0 fluorometer (Qubit dsDNA HS Assay kit; Q32851, Thermo Fisher Scientific) and an Advanced Analytical FEMTO Pulse instrument (Genomic DNA 165 kb Kit; FP-1002-0275, Agilent), respectively. Further steps included removal of single strand overhangs, DNA damage repair, end-repair & A tailing, ligation of barcoded overhang adapters, and then purification of the library using AMPure PB beads. The libraries were quality controlled using the steps described above and then pooled using the PacBio microbial multiplexing calculator (PacBio guidelines 2020). Prior to and after size selection, the library pool was purified using AMPure PB beads. Size selection was performed with the BluePippin instrument (Sage Science; BLU0001) using BluePippin with dye-free, 0.75% Agarose Cassettes and S1 Marker (Sage Science; BLF7510) wherein the selection cut-off was set at 6000 bp. Library pool concentration and size was again assessed using a Thermo Fisher Scientific Qubit 4.0 flurometer and an Advanced Analytical FEMTO Pulse instrument (as described above), respectively. The final library pools were on average 11.4 Kb in size. PacBio Sequencing primer v4 and Sequel DNA Polymerase 3.0 were annealed and bound, respectively, to the DNA template libraries. The polymerase binding time was 1 h and the complex was cleaned using 1.2 X AMPure PB beads. The libraries were loaded at an on-plate concentration of 10 or 11 pM using diffusion loading, along with the use of Spike-In internal control. SMRT sequencing was performed in CLR sequencing mode on the Sequel System with Sequel Sequencing kit 3.0, SMRT Cells 1M v3, a 2h pre-extension followed by a 600 min movie time and via PacBio SMRT Link v8.0. Likewise, the second extraction, preparation of the library, and PacBio sequencing were performed by the Next Generation Sequencing Platform (University of Bern, Switzerland).

### ONT long read sequencing

For ONT long read sequencing, the gDNAs extracted using the GenElute™ bacterial genomic DNA (Sigma-Aldrich) were treated with the Short Read Removal Kit XS, (#SKU SS-100-121-01; Circulomics Inc., Baltimore, US) according to the manufacturer’s protocol. ONT libraries were prepared using sequencing kit SQK-LSK109 according to the manufacturer’s protocol (using doubled incubation time) and sequenced in-house (Molecular Diagnostics, Genomics and Bioinformatics, Wädenswil, Switzerland) on a MinION Flow Cell R9.4.1 (1 Flow Cell per strain). Fast5 files from ONT sequencing were obtained with ONT MinKNOW Core software v3.6.5 (default quality filtering of Q7 applied); the base-calling step was carried out with Guppy v4.4.2.

## Genome Assembly

Illumina paired-end reads were trimmed with Trimmomatic v0.39 (Bolger et al., 2014) (parameters: -phred 33 leading:3 trailing:3 sildingwindow:4:15 minlen:36) using FastQC v0.11.9 (Andrews, 2010) to inspect the read quality before and after trimming (Andrews, 2010). The majority of assemblies were generated using PacBio long reads and the Flye assembler (Kolmogorov et al., 2019). For very complex, repeat-rich assemblies, additionally ONT MinION long read data and/or Trycycler (Wick et al., 2021a) were used to generate a consensus assembly and circularize the assemblies. One strain was assembled using the hybrid assembler Unicycler (Wick et al., 2017) instead of Flye. A deeper focus on the different strategies for the assembling of the different type strains can be found below. Table S2 provides an overview of all assembled type strains.

### Assembly strategies for the different type strains

#### Strains 02, 04, 06, 07, 08, 09, 11 (Table S2, Table S3)

Raw PacBio sequence reads were filtered according to quality and length using filtlong v0.2.0 (https://github.com/rrwick/Filtlong) with Illumina paired end libraries as external reference. The filtered reads were *de novo* assembled using Flye v2.8.1-b1676 (Kolmogorov et al., 2019) with default parameters (except for using 3 polishing iterations; in addition, the minimum overlap for Flye and the filtlong parameters were adapted to each respective data set; for more detail, see Supplementary Table S3). Different parameter settings were applied, and the best assembly was chosen after manual evaluation considering assembly graph, contig connectivity & circularity, aiming to avoid unresolved regions and artificial repeats. The start position of the assembly was manually adjusted to the *dnaA* gene, which was identified using NCBI’s Prokaryotic Genome Annotation Pipeline (PGAP) (version 2021-01-11.build5132) (Tatusova et al., 2016). The assemblies were polished with long reads using PacBio’s tool pbmm2 v1.4.0 (https://github.com/PacificBiosciences/pbmm2/) for alignment with default parameters (except: minimum length set to 1000) and gcpp v1.9.0 (https://github.com/PacificBiosciences/gcpp) for consensus sequence generation.

#### Strains 01, 03, 12, 13 (Table S2)

Due to long repeats present in these four strains, PacBio reads could not fully resolve the genomes and additional very long ONT sequence reads were obtained. Due to the huge library size, the ONT reads were first subsampled using rasusa v 0.3.0 (Hall 2019) to generate 25 subread sets, each set containing enough reads to achieve 200x genome coverage. Each subread set was filtered using filtlong v0.2.0 according to quality and length (default parameters, except: minimum subread length of 1000, keep percent 95) and *de novo* assembled using Flye v2.8.1b1676, resulting in 25 draft assemblies. Next, Trycycler v0.5.0 (Wick 2021b) was used to generate a consensus assembly as described in https://github.com/rrwick/Trycycler/wiki/How-to-run-Trycycler.

#### Strains 05, 10, 15 (Table S2, Table S4)

No single run with the Flye assembler was able to fully resolve and circularize the genomes of these strains. Therefore, assembly parameters (read quality and read length using filtlong, and minimum overlap length (--min-overlap) for Flye) were varied to create different linear or fragmented assemblies (for more details, see Supplementary Table S4). Trycycler was used to generate circular consensus assemblies using the fragmented assemblies as input.

#### Strain 14 (Table S2)

Due to issues with the Flye assemblies (incorrect and inconsistent plasmid assembly likely due to long repeats shared between plasmids and chromosome), a hybrid assembly was performed using Unicycler v0.4.8 (Wick et al., 2017) with unfiltered PacBio reads and trimmed 2 × 150 bp Illumina reads. This approach was able to resolve the complete genome sequence.

## Short read polishing and assembly quality control

All assemblies were further polished using trimmed 2 × 150 bp Illumina reads and Freebayes v.1.3.2 (Garrison et al., 2012) (minimum alternate fraction, 0.5; minimum alternate count, 5). Variants were manually inspected in the Integrated Genome Viewer (IGV) (Thorvaldsdóttir et al., 2013) and subsequently corrected with bcftools v.1.10.2 (Danecek et al., 2021) to adjust any potentially remaining small sequencing errors and systematic long-read sequencing errors such as indels in homopolymer regions.

To detect potentially missing short plasmids in the long read-based assemblies, PlasmidSpades v3.13.1 (Antipov et al., 2016) was run to assemble the Illumina short reads. If a plasmid was detected, its sequence was polished with Illumina short reads as described above and added to the final assembly (Supplementary Table S2).

To verify the circularity and completeness of the *de novo* assemblies, long reads (from PacBio or ONT, respectively) and Illumina reads were mapped to each assembly using minimap2 and bwa-mem v0.7.17, respectively (with default parameters, before further inspecting the alignments in IGV) (Li 2018).

Mapping quality of the reads was assessed using qualimap v.2.2.1 (Okonechnikov et al., 2016). The completeness of the final assembly was further evaluated using the benchmarking universal single-copy orthologous genes (BUSCO) software v5.0.0 (Seppey et al, 2019). An in-house prototype for the detection of repeats was run to identify large repeats (some of which could indicate misassembled regions) as a final QC step (Schmid et al., 2018).

The finished genomes were annotated using a local installation of NCBI’s prokaryotic genome annotation pipeline (PGAP) (version 2021-01-11.build5132) (Tatusova et al., 2016).

## Phenotypic Antimicrobial Susceptibility Testing

For all 350 isolates selected amount the 23 predominant species, the respective minimum inhibitory concentrations (MIC) were determined with microdilution assays using the Microscan System (Beckman Coulter Microbiology, West Sacramento, CA), according to the manufacturer's instructions. The choice of the different antibiotics panels was based on the characteristics of the different bacterial species.

For the *Staphylococcus* species, *Mammaliicoccus* species, and *Bacillus cereus* group, the MIC for 30 different antibiotics were determined using the Gram positive MIC panel “Pos MIC 32” (Beckman Coulter), which included the following antimicrobial agents: amoxacillin/ K clavulanate, ampicillin, azithromycin, cefepime, cefotaxime, cefuroxime, chloramphenicol, ciprofloxacin, clindamycin, daptomycin, ertapenem, erythromycin, fosfomycin, fusic acid, gentamicin, imipenem, levofloxacin, linezolid, meropenem, moxifloxacin, nitrofurantoin, oxacillin, penicillin, rifampin, synercid, teicoplanin, tetracycline, tobramycin, trimethoprim/sulfamethoxazole, and vancomycin. To determine the MIC of the antimicrobials, the breakpoints were assigned according to the range published in the 2022 guidelines of the European Committee on Antimicrobial Susceptibility Testing (EUCAST) (EUCAST, 2022). When no range was available for an antibiotic included in the panels by the EUCAST 2022 guidelines, the breakpoints were defined based on the range described by the Clinical and Laboratory Standards Institute (CLSI 2012).

For the *Mammaliicoccus* spp*.* and *Bacillus cereus group* we used the same breakpoint as for the *Staphylococcus* spp. For *Aerococcus viridans* and *Lactococcus* spp., the “MicroStrep plus® Type 6” panel (Beckman Coulter) was used. For *Arthrobacter gandavensis*, *Enterococcus* spp., and *Streptococcus* spp., the “Pos MIC E 37” panel (Beckman Coulter) was used while for the Gram-negative *Acinetobacter lwoffii* and *Escherichia coli* the “Neg MIC 44” panel (Beckman Coulter) was used.

## References

Andrews, S. (2010) FastQC:  A Quality Control Tool for High Throughput Sequence Data [Online]. Available online at: http://www.bioinformatics.babraham.ac.uk/projects/fastqc/.

Antipov D, Hartwick N, Shen M, Raiko M, Lapidus A, Pevzner PA. (2016) plasmidSPAdes: assembling plasmids from whole genome sequencing data. *Bioinformatics.* Nov 15;32(22):3380-3387. doi: 10.1093/bioinformatics/btw493.

CLSI, Performance Standards for Antimicrobial Susceptibility Testing; Twenty-Second Informational Supplement. CLSI document M100-S22. Wayne, PA: Clinical Laboratory Institute; 2012.

Danecek P, Bonfield JK, Liddle J, Marshall J, Ohan V, Pollard MO, Whitwham A, Keane T, McCarthy SA, Davies RM, Li H. (2021) Twelve years of SAMtools and BCFtools. Gigascience. Feb 16;10(2): giab008. doi: 10.1093/gigascience/giab008.

EUCAST, The European Committee on Antimicrobial Susceptibility Testing. Breakpoint tables for interpretation of MICs and zone diameters. Version 12.0, 2022. http://www.eucast.org.

Garrison E, Marth G. (2012) Haplotype-based variant detection from short-read sequencing. arXiv preprint arXiv:1207.3907 [q-bio.GN], https://arxiv.org/abs/1207.3907.

Hall, Michael B. (2019, November 16). Rasusa: Randomly subsample sequencing reads to a specified coverage (Version 0.1.0). Zenodo. http://doi.org/10.5281/zenodo.3546168.

Kolmogorov M, Yuan J, Lin Y, Pevzner PA. (2019) Assembly of long, error-prone reads using repeat graphs. *Nat Biotechnol*. May;37(5):540-546. doi: 10.1038/s41587-019-0072-8.

Li H. (2018) Minimap2: pairwise alignment for nucleotide sequences. *Bioinformatics.* Sep 15;34(18):3094-3100. doi: 10.1093/bioinformatics/bty191.

Okonechnikov K, Conesa A, García-Alcalde F. (2016) Qualimap 2: advanced multi-sample quality control for high-throughput sequencing data*. Bioinformatics*. Jan 15;32(2):292-4. doi: 10.1093/bioinformatics/btv566.

PacBio guideline, (2020), *Procedure & Checklist – Preparing Multiplexed Microbial Libraries Using SMRTbell® Express Template Prep Kit 2.0*" - Part Number 101-696-100 Version 06, https://www.pacb.com/wp-content/uploads/Procedure-Checklist-–-Preparing-Multiplexed-Microbial-Libraries-Using-SMRTbell-Express-Template-Prep-Kit-2.0.pdf. (Accessed March 1, 2020).

Pitcher DG, Sauders NA, Owen RJ. (1989) Rapid extraction of bacterial genomic DNA with guanidium thiocyanate. *Lett Appl Microbiol*, 8(4): 151-156, doi:10.1111/j.1472-765X.1989.tb00262.x.

Schmid M, Frei D, Patrignani A, Schlapbach R, Frey JE, Remus-Emsermann MNP, Ahrens CH. (2018) Pushing the limits of *de novo* genome assembly for complex prokaryotic genomes harboring very long, near identical repeats. *Nucleic Acids Res*. Sep 28;46(17):8953-8965. doi: 10.1093/nar/gky726.

Seppey M., Manni M., Zdobnov E.M. (2019) BUSCO: Assessing Genome Assembly and Annotation Completeness. In: Kollmar M. (eds) Gene Prediction. Methods in Molecular Biology, vol 1962. Humana, New York, NY. doi.org/10.1007/978-1-4939-9173-0_14.

Tatusova T, DiCuccio M, Badretdin A, Chetvernin V, Nawrocki EP, Zaslavsky L, Lomsadze A, Pruitt KD, Borodovsky M, Ostell J. (2016) NCBI prokaryotic genome annotation pipeline. Nucleic Acids Res. Aug 19;44(14):6614-24. doi: 10.1093/nar/gkw569.

Thorvaldsdóttir H, Robinson JT, Mesirov JP. (2013) Integrative Genomics Viewer (IGV): high-performance genomics data visualization and exploration. *Brief Bioinform*. Mar;14(2):178-92. doi: 10.1093/bib/bbs017.

Wick RR, Judd LM, Gorrie CL, Holt KE. (2017) Unicycler: Resolving bacterial genome assemblies from short and long sequencing reads. *PLoS Comput Biol*. Jun 8;13(6): e1005595. doi: 10.1371/journal.pcbi.1005595.

Wick RR, Judd LM, Cerdeira LT, Hawkey J, Méric G, Vezina B, Wyres KL, Holt KE. (2021a) Trycycler: consensus long-read assemblies for bacterial genomes. *Genome Biol.* Sep 14;22(1):266. doi: 10.1186/s13059-021-02483-z.

Wick RR. (2021b, March 19). rrwick/Trycycler: Trycycler v0.5.0 (Version v0.5.0). Zenodo. http://doi.org/10.5281/zenodo.4620349

# Supplementary Tables

**Table S1**: List of eight type strains with available complete genome sequence downloaded from the NCBI. The table displays the different identifiers and accession numbers of these eight type strains.

| **Species** | **Strain** | **N. DSMZ**  **Type**  **strain** | **Biosample** | **Bioproject** | **Assembly** |
| --- | --- | --- | --- | --- | --- |
| *Staphylococcus xylosus* | NCTC11043 | 20266 | SAMEA3539705 | PRJEB6403 | GCA_900458755.1 |
| *Mammaliicoccus sciuri* | NCTC12103 | 20345 | SAMEA3505362 | PRJEB6403 | GCA_900474615.1 |
| *Staphylococcus aureus* | NCTC8532 | 20231 | SAMEA2479567 | PRJEB6403 | GCA_900706775.1 |
| *Bacillus cereus* | ATCC 14579 | 31 | SAMN10591533 | PRJNA509739 | GCA_006094295.1 |
| *Bacillus thuringiensis* | ATCC 10792 | 2046 | SAMN00738287 | PRJNA29723 | GCA_000161615.1 |
| *Escherichia coli* | ATCC 11775 | 30083 | SAMN10252913 | PRJNA472652 | GCA_003697165.2 |
| *Streptococcus agalactiae* | NCTC8181 | 2134 | SAMEA3696441 | PRJEB6403 | GCA_900458965.1 |
| *Streptococcus uberis* | NCTC3858 | 20569 | SAMEA3871780 | PRJEB6403 | GCA_900475595.1 |

Table S2: List of 15 type strains for which the complete genome sequence was assembled *de novo*. A combination of long reads from the PacBio or ONT platforms and Illumina short reads was used. The accession numbers can be found in the NCBI Bioproject PRJNA936091.

| **Strain** | **Organism** | **Chromosomes and plasmids** | **Length**  **(bp)** | **GC**  **Content**  **(%)** | **Coverage**  **(illumina)** | **Coverage**  **(PacBio, ONT)** | **Long read sequencing technology** |
| --- | --- | --- | --- | --- | --- | --- | --- |
| 01 | *Staphylococcus equorum* | Chromosome_1  Plasmid_1  Plasmid_2  Plasmid_3  Plasmid_4  Plasmid_5 | 2’698’219  28’553  4’846  4’397  2’361  2’216 | 33.1 29.9 30.3 31.6 29.3  29.0 | 638x | 5351x | ONT |
| 02 | *Staphylococcus haemolyticus* | Chromosome_1  Plasmid_1  Plasmid_2 | 2’516’900  28’019  26’108 | 32.9 33.5  30.1 | 533x | 686x | PacBio |
| 03 | *Staphylococcus succinus* | Chromosome_1  Plasmid_1  Plasmid_2 | 2’757’983  45’941  27’211 | 33.1 31.6  31.2 | 461x | 5072x | ONT |
| 04 | *Mammaliicoccus vitulinus* | Chromosome_1  Plasmid_1 | 2’643’161  4’440 | 32.7  30.1 | 297x | 299x | PacBio |
| 05 | *Staphylococcus warneri* | Chromosome_1 | 2’428’398 | 32.8 | 496x | 642x | PacBio |
| 06 | *Arthrobacter gandavensis* | Chromosome_1 | 3’576’047 | 65.5 | 404x | 320x | PacBio |
| 07 | *Lactococcus*  *lactis* | Chromosome_1  Plasmid_1  Plasmid_2 | 2’518’868  58’341  13’250 | 35.4 34.8  32.1 | 649x | 288x | PacBio |
| 08 | *Acinetobacter lwoffii* | Chromosome_1  Plasmid_1  Plasmid_2  Plasmid_3  Plasmid_4  Plasmid_5 | 3’166’595  221’373  55’306  11’681  5’879  2’845 | 43.3 40.5 39.1 36.4  36.7  33.0 | 413x | 298x | PacBio |
| 09 | *Enterococcus faecalis* | Chromosome_1 | 2’866’949 | 37.6 | 571x | 647x | PacBio |
| 10 | *Bacillus mycoides* | Chromosome_1  Plasmid_1  Plasmid_2  Plasmid_3 | 5’257’667  361’290  10’361  9’935 | 35.5 33.9 31.4  32.6 | 69x | 153x | PacBio |
| 11 | *Enterococcus saccharolyticus* | Chromosome_1 | 2’622’437 | 37.0 | 142x | 340x | PacBio |
| 12 | *Enterococcus faecium* | Chromosome_1  Plasmid_1 | 2’529’607  142’622 | 38.2  36.3 | 514x | 12307x | ONT |
| 13 | *Staphylococcus chromogenes* | Chromosome_1  Plasmid_1 | 2’280’008  20’779 | 36.8  29.0 | 635x | 664x | ONT |
| 14 | *Lactococcus garviae* | Chromosome_1  Plasmid_1  Plasmid_2 | 2’048’553  34’568  10’137 | 38.7 34.3  34.0 | 766x | 143x | PacBio |
| 15 | *Aerococcus viridans* | Chromosome_1 | 2’204’952 | 39.4 | 392x | 302x | PacBio |

Table S3: Final parameters used for filtlong and Flye to create complete assemblies for strains 02, 04, 06, 07, 08, 09, 11 (Table S2).

| **Strain** | **min_length (filtlong)** | **Keep_percent (filtong)** | **Min_overlap (Flye)** |
| --- | --- | --- | --- |
| 02 | 8000 | 70 | 5000 |
| 04 | 5000 | 90 | 1000 |
| 06 | 3000 | 90 | Auto (5000) |
| 07 | 3000 | 90 | 3000 |
| 08 | 2000 | 90 | 3000 |
| 09 | 2000 | 50 | 2000 |
| 11 | 500 | 90 | 7000 |

Table S4: Parameters used for filtlong and Flye and assembly information for all input assemblies used by Trycycler to create consensus assemblies for Strains 05, 10 and 15 (Table S2).

| **Strain** | **Min_length (filtlong)** | **Keep_ percent (filtlong)** | **Min_Overlap (Flye)** | **# of contigs** | **Contig length** | **Circular** |
| --- | --- | --- | --- | --- | --- | --- |
| 05 | 1000 | 35 | 2000 | 3 | 1049065, 945808, 486196 | N,N,N |
|  | 1000 | 90 | 6000 | 2 | 964401, 928695 | N,N |
|  | 2000 | 70 | 2000 | 1 | 2428309 | N |
|  | 7000 | 70 | Auto | 2 | 2426405, 30167 | N,N |
|  | 1000 | 70 | 2000 | 1 | 2428301 | N |
|  | 2000 | 35 | Auto | 3 | 1049066, 945807, 486188 | N,N,N |
|  | 7000 | 50 | 2000 | 1 | 2428314 | N |
|  | 9000 | 90 | 6000 | 2 | 2474474, 14965 | N,N |
| 10 | 500 | 90 | 1000 | 5 | 5054957, 361287, 199246, 20722, 9934 | N,Y,N,Y,  Y |
|  | 500 | 90 | 2000 | 5 | 5054963, 361278, 199246, 20722, 19870 | N,Y,N,Y,  Y |
|  | 8000 | 90 | 1000 | 4 | 5300849, 361285, 19870, 10360 | N,Y,Y,Y |
| 15 | 10000 | 95 | 1000 | 1 | 2201962 | N |
|  | 10000 | 95 | 5000 | 1 | 2201895 | N |
|  | 1000 | 95 | 5000 | 2 | 1876748, 321891 | N,N |
|  | 11000 | 95 | 1000 | 1 | 2202721 | N |
|  | 12000 | 95 | 2000 | 1 | 2253234 | N |
|  | 12000 | 95 | 1000 | 1 | 2229151 | N |
|  | 3000 | 95 | 2000 | 1 | 2204268 | N |
|  | 8000 | 90 | 1000 | 1 | 2202718 | N |
|  | 8000 | 95 | 5000 | 1 | 2201934 | N |
|  | 8000 | 95 | 1000 | 1 | 2202718 | N |
|  | 9000 | 95 | 1000 | 1 | 2202723 | N |
|  | 9000 | 95 | 5000 | 1 | 2201961 | N |

**Table 5S**: Descriptive data regarding the cows involving in the study. The cows age, stage and number of lactations were included.

|  | **Farm 1** | | | **Farm 2** | | | **Farm 3** | | | **Farm 4** | | | **Farm 5** | | | **Farm 6** | | **Farm 7** | | | **Farm 8** | | | **Farm 9** | | | **Total (N.)** | **Percentage (%)** |
| --- | --- | --- | --- | --- | --- | --- | --- | --- | --- | --- | --- | --- | --- | --- | --- | --- | --- | --- | --- | --- | --- | --- | --- | --- | --- | --- | --- | --- |
| Sampling | **0** | **1** | **2** | **0** | **1** | **2** | **0** | **1** | **2** | **0** | **1** | **2** | **0** | **1** | **2** | **0** | **1** | **0** | **1** | **2** | **0** | **1** | **2** | **0** | **1** | **2** |  |  |
| N. cows sampled | 11 | 10 | 10 | 9 | 8 | 9 | 10 | 10 | 10 | 10 | 10 | 10 | 10 | 10 | 10 | 9 | 10 | 10 | 10 | 10 | 10 | 10 | 10 | 10 | 10 | 10 | 256 |  |
| Average age cows (year) | 5.3 | 4.8 | 4.6 | 4.5 | 4.6 | 5.1 | 6.2 | 6.8 | 8.5 | 5.6 | 6 | 6.8 | 4.4 | 4.7 | 5.5 | 8.6 | 8.9 | 7.4 | 7.8 | 7.9 | 4.8 | 5.1 | 5.3 | 6.4 | 5.9 | 6.5 |  |  |
| Average age cows (days) | 1949 | 1785 | 1696 | 1663 | 1708 | 1863 | 2295 | 2491 | 3129* | 2052 | 2201 | 2487 | 1618 | 1745 | 2014 | 3149 | 3263 | 2710 | 2864 | 2904 | 1765 | 1884 | 1961 | 2370 | 2185 | 2390 |  |  |
| Stage of lactation, early | 3 | 1 | 1 | 3 | 0 | 3 | 3 | 0 | 2 | 3 | 2 | 4 | 10 | 0 | 8 | 0 | 3 | 2 | 2 | 4 | 5 | 0 | 5 | 5 | 0 | 1 | 70 | 27 |
| Stage of lactation, mid | 3 | 3 | 3 | 2 | 2 | 4 | 2 | 4 | 4 | 4 | 0 | 4 | 0 | 9 | 1 | 3 | 0 | 5 | 1 | 3 | 2 | 5 | 1 | 3 | 1 | 7 | 76 | 30 |
| Stage of lactation, late | 5 | 6 | 6 | 4 | 6 | 2 | 4 | 6 | 4 | 3 | 8 | 2 | 0 | 1 | 1 | 6 | 7 | 3 | 6 | 3 | 3 | 5 | 4 | 2 | 9 | 2 | 108 | 42 |
| Stage of lactation (ND) |  |  |  |  |  |  | 1 |  |  |  |  |  |  |  |  |  |  |  | 1 |  |  |  |  |  |  |  |  |  |
| Lactation number 1 | 2 | 6 | 6 | 4 | 3 | 1 | 2 | 2 | 3 | 1 | 1 | 0 | 3 | 3 | 3 | 0 | 0 | 2 | 0 | 1 | 3 | 3 | 2 | 1 | 3 | 0 | 55 | 21 |
| Lactation number 2 and 3 | 5 | 1 | 2 | 3 | 4 | 5 | 2 | 4 | 2 | 6 | 5 | 2 | 6 | 6 | 4 | 1 | 1 | 2 | 3 | 2 | 4 | 5 | 5 | 2 | 4 | 3 | 89 | 35 |
| Lactation number > 3 | 4 | 3 | 2 | 2 | 1 | 3 | 5 | 4 | 5 | 3 | 4 | 8 | 1 | 1 | 3 | 8 | 9 | 6 | 6 | 7 | 3 | 2 | 3 | 7 | 3 | 7 | 110 | 43 |
| Lactation number (ND) |  |  |  |  |  |  | 1 |  |  |  |  |  |  |  |  |  |  |  | 1 |  |  |  |  |  |  |  |  |  |

Referring to the main text, considering the stage of lactation, we referred to three different stages divided in early (14-100 days after calving), mid (100-200 days after calving), and late lactation (>200 days after calving). For lactation number, we divided the cows into three different groups: i) 1^st^ lactation (primiparous), ii) 2^nd^ and 3^rd^ lactation, and iii) >3 lactations.

Table S6: Illumina sequencing data (350 isolates) and overview of selected parameters including coverage and median length for the total chromosomal sequences.

| Species | N. of strains | Seq. technology | Assembly method | Median coverage | Min coverage | Max coverage | Median total sequence length |
| --- | --- | --- | --- | --- | --- | --- | --- |
| *Staphylococcus xylosus* | 101 | HiSeq Illumina | SeqMan NGen 16 software | 551x | 287x | 873x | 2'755'619 |
| *Mammaliicoccus sciuri* | 83 | HiSeq Illumina | SeqMan NGen 16 software | 585x | 120x | 894x | 2'814'906 |
| *Staphylococcus succinus* | 22 | HiSeq Illumina | SeqMan NGen 16 software | 522x | 157x | 812x | 2'763'069 |
| *Staphylococcus equorum* | 16 | HiSeq Illumina | SeqMan NGen 16 software | 519x | 461x | 1013x | 2'706'513 |
| *Staphylococcus aureus* | 9 | HiSeq Illumina | SeqMan NGen 16 software | 570x | 496x | 665x | 2'710'704 |
| *Mammaliicoccus vitulinus* | 9 | HiSeq Illumina | SeqMan NGen 16 software | 615x | 510x | 667x | 2'649'562 |
| *Staphylococcus warneri* | 5 | HiSeq Illumina | SeqMan NGen 16 software | 515x | 480x | 602x | 2'431'154 |
| *Staphylococcus haemolyticus* | 3 | HiSeq Illumina | SeqMan NGen 16 software | 520x | 289x | 645x | 2'522'048 |
| *Staphylococcus chromogenes* | 3 | HiSeq Illumina | SeqMan NGen 16 software | 656x | 631x | 677x | 2'286'148 |
| *Acinetobacter lwoffii* | 2 | HiSeq Illumina | SeqMan NGen 16 software | 742x | 633x | 850x | 3'173'634 |
| *Aerococcus viridans* | 9 | HiSeq Illumina | SeqMan NGen 16 software | 658x | 588x | 712x | 2'208'760 |
| *Arthrobacter gandavensis* | 2 | HiSeq Illumina | SeqMan NGen 16 software | 287x | 264x | 309x | 3'508'622 |
| *Bacillus cereus group* | 35 | HiSeq Illumina | SeqMan NGen 16 software | 236x | 118x | 457x | 5'424’088 |
| *Enterococcus faecalis* | 1 | HiSeq Illumina | SeqMan NGen 16 software | 803x |  |  | 2'876'355 |
| *Enterococcus faecium* | 2 | HiSeq Illumina | SeqMan NGen 16 software | 512x | 365x | 669x | 2'533'543 |
| *Enterococcus saccharolyticus* | 6 | HiSeq Illumina | SeqMan NGen 16 software | 835x | 737x | 889x | 2'630'767 |
| *Escherichia coli* | 13 | HiSeq Illumina | SeqMan NGen 16 software | 353x | 286x | 546x | 4'908'422 |
| *Lactococcus garvieae* | 7 | HiSeq Illumina | SeqMan NGen 16 software | 651x | 489x | 877x | 2'051'820 |
| *Lactococcus lactis* | 7 | HiSeq Illumina | SeqMan NGen 16 software | 578x | 431x | 664x | 2'525'684 |
| *Streptococcus agalactiae* | 11 | HiSeq Illumina | SeqMan NGen 16 software | 774x | 525x | 1082x | 2'255'253 |
| *Streptococcus uberis* | 4 | HiSeq Illumina | SeqMan NGen 16 software | 774x | 746x | 816x | 1'980'421 |

**Table S10**: Overview of the isolates that carried the *tetK* gene. All reads of isolates positive for the *tetK* gene were assembled with the closed plasmid as references and compared with Clone Manager 9.51. The table showed the 31 isolates plasmids, the characteristics of length of the sequences and similarity with the reference plasmid, biosample SAMN29790278.

| **NCBI_biosample_accession** | **Species** | **Farm** | **Length (bp)** | **Similarity (%)** | ***rep* gene** |
| --- | --- | --- | --- | --- | --- |
| SAMN29790278 (Ref. plasmid) | *Staph. xylosus* | Farm 1 | 4440 | 100.0 | *rep7a* |
| SAMN29790288 | *Staph. xylosus* | Farm 1 | 4666 | 96.9 | *rep7a* |
| SAMN29790292 | *Staph. xylosus* | Farm 2 | 4440 | 99.2 | *rep7a* |
| SAMN29790395 | *Mamm. sciuri* | Farm 2 | 4448 | 99.0 | *rep7a* |
| SAMN29790396 | *Mamm. sciuri* | Farm 2 | 4440 | 100.0 | *rep7a* |
| SAMN29790294 | *Staph. xylosus* | Farm 2 | 4440 | 99.7 | *rep7a* |
| SAMN29790295 | *Staph. xylosus* | Farm 2 | 4440 | 99.6 | *rep7a* |
| SAMN29790400 | *Mamm. sciuri* | Farm 2 | 4439 | 99.7 | *rep7a* |
| SAMN29790298 | *Staph. xylosus* | Farm 2 | 4439 | 99.7 | *rep7a* |
| SAMN29790299 | *Staph. xylosus* | Farm 2 | 4439 | 99.7 | *rep7a* |
| SAMN29790301 | *Staph. xylosus* | Farm 2 | 4439 | 99.7 | *rep7a* |
| SAMN29790302 | *Staph. xylosus* | Farm 2 | 4439 | 99.7 | *rep7a* |
| SAMN29790303 | *Staph. xylosus* | Farm 2 | 4440 | 99.7 | *rep7a* |
| SAMN29790327 | *Staph. xylosus* | Farm 4 | 4440 | 100.0 | *rep7a* |
| SAMN29790328 | *Staph. equorum* | Farm 4 | 4440 | 100.0 | *rep7a* |
| SAMN29790491 | *Staph. xylosus* | Farm 5 | 4444 | 98.9 | *rep7a* |
| SAMN29790437 | *Mamm. sciuri* | Farm 5 | 4440 | 98.6 | *rep7a* |
| SAMN29790334 | *Staph. xylosus* | Farm 5 | 4440 | 99.7 | *rep7a* |
| SAMN29790336 | *Staph. xylosus* | Farm 5 | 4440 | 99.7 | *rep7a* |
| SAMN29790337 | *Staph. xylosus* | Farm 5 | 4440 | 99.6 | *rep7a* |
| SAMN29790338 | *Staph. xylosus* | Farm 5 | 4440 | 99.7 | *rep7a* |
| SAMN29790339 | *Staph. xylosus* | Farm 5 | 4440 | 99.7 | *rep7a* |
| SAMN29790340 | *Staph. xylosus* | Farm 5 | 4440 | 99.7 | *rep7a* |
| SAMN29790343 | *Staph. xylosus* | Farm 5 | 4440 | 99.7 | *rep7a* |
| SAMN29790346 | *Staph. xylosus* | Farm 5 | 4440 | 99.7 | *rep7a* |
| SAMN29790347 | *Staph. xylosus* | Farm 5 | 4440 | 99.9 | *rep7a* |
| SAMN29790515 | *Staph. warneri* | Farm 7 | 4440 | 99.7 | *rep7a* |
| SAMN29790361 | *Staph. xylosus* | Farm 7 | 4440 | 99.7 | *rep7a* |
| SAMN29790429 | *Mamm. sciuri* | Farm 8 | 4440 | 98.7 | *rep7a* |
| SAMN29790375 | *Staph. xylosus* | Farm 9 | 4438 | 97.7 | *rep7a* |
| SAMN29790372 | *Staph. xylosus* | Farm 9 | 4435 | 98.0 | *rep7a* |

**Supplementary Figure**

**
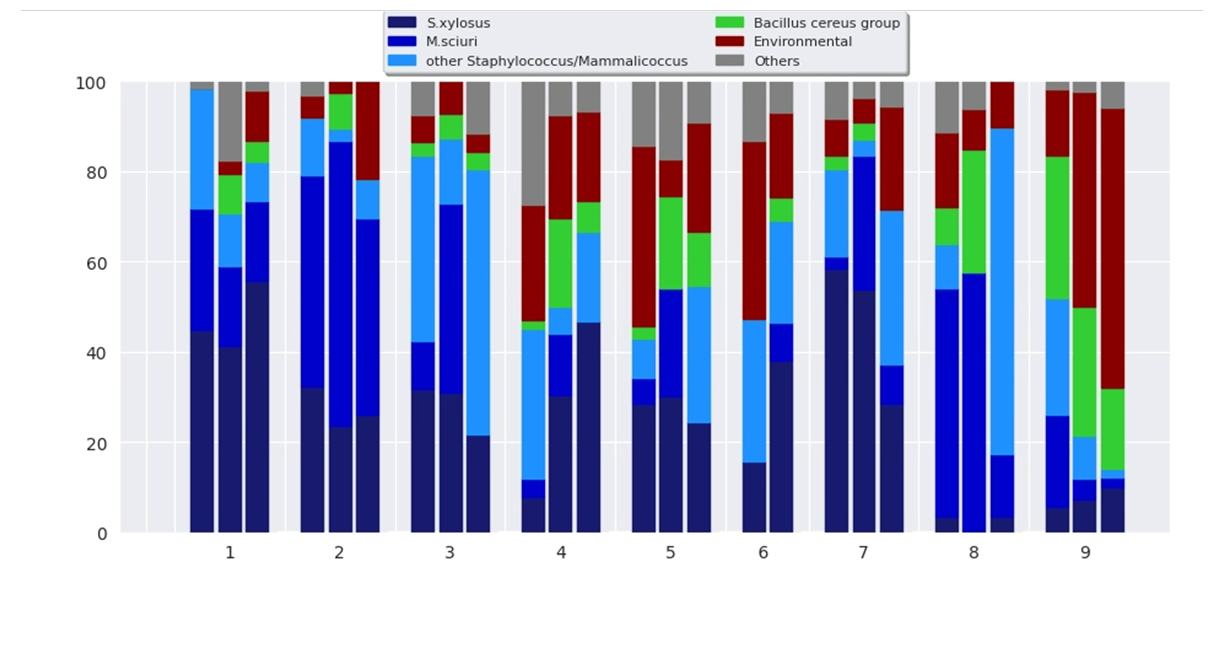
**

**Figure S1:** Distribution of the different bacteria/groups during sampling time (T0, T1, T2).


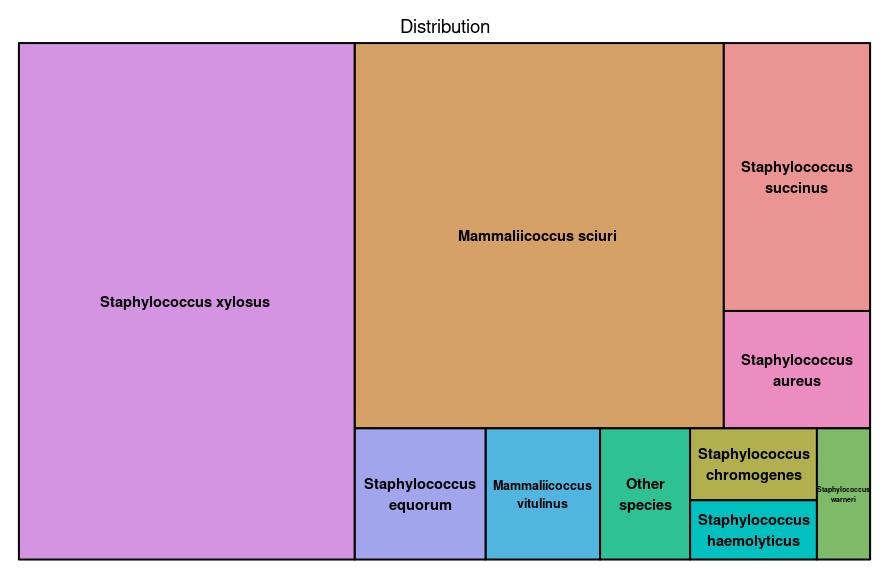


# Figure S2: Distribution of the different *Staphylococcus* spp.

The figure presents an overview of the relative distribution of the respective bacterial species that were identified in our intramammary bacteriome study (created with R v 4.0.05). The total number of the non-*aureus* staphylococci and mammaliicocci (NASM) and the *Staphylococcus aureus* isolates amounted to 819; their respective percentage is shown below:

- *Staphylococcus xylosus* (n=323, 39.4%)
- *Mammaliicoccus sciuri* (n=265, 32.4%)
- *Staphylococcus succinus* (n=73, 8.9%)
- *Staphylococcus equorum* (n=32, 3.9%)
- *Staphylococcus aureus* (n=32, 3.9%)
- *Mammaliicoccus vitulinus* (n=28, 3.4%)
- *Staphylococcus chromogenes* (n=17, 2.1%)
- *Staphylococcus haemolyticus* (n=15, 1.8%)
- *Staphylococcus warneri* (n=13, 1.6%)
- Other species (n=21, 2.6%)

Supplementary Data

Our manually curated database of *Staphylococcus* spp. ARGs (105 fasta genes) is released as Supplementary Word File.
